# CD8+ T cell activation in endometrial cancer: prognostic implications and potential for personalized therapy

**DOI:** 10.3389/fimmu.2025.1542669

**Published:** 2025-04-28

**Authors:** HaoTong Guan, QiuShuang Xiong, JiaQiang Xiong, Yanyan Liu, Wei Zhang

**Affiliations:** ^1^ Department of Gynecologic, Zhongnan Hospital of Wuhan University, Wuhan, Hubei, China; ^2^ Wuhan Union Hospital, Tongji Medical College, Huazhong University of Science and Technology, Wuhan, Hubei, China

**Keywords:** endometrial cancer (EC), immunotherapy, immune microenvironment (IME), CD8+ T cell, personalized treatment

## Abstract

**Background:**

As an important component in preventing the progression of endometrial cancer, CD8 T cells play a crucial role in this process and are important targets for immunotherapy. However, the status of CD8+ T cells in endometrial cancer and the key genes influencing their activation still remain to be elucidated.

**Methods:**

Genes associated with the activation of CD8+ T cells were identified through differential analysis and weighted gene co-expression network analysis (WGCNA). A risk score model was constructed using the least absolute shrinkage and selection operator (LASSO) and multivariate Cox regression. The clinical characteristics and differences between the high-risk group and the low-risk group were explored, and the applicability of the model to chemotherapy, poly (ADP-ribose) polymerase (PARP) inhibitors, and immune checkpoint inhibitors was evaluated. The characteristics of the model at the single-cell level were studied, and the tumor-suppressive effect of ASB2 was verified through experiments on endometrial cancer cells.

**Results:**

A risk model based on genes related to the activation of CD8+ T cells was constructed, and the prognostic differences were verified using the Kaplan-Meier curve. A nomogram was designed to predict the survival probability. Pathway analysis showed that it was related to metabolism and DNA repair. There were significant differences between the high-risk and low-risk groups in terms of tumor mutational burden (TMB), checkpoint molecules, and major histocompatibility complex (MHC) class I molecules, and they had different sensitivities to different therapies. The tumor-suppressive effect of ASB2 was confirmed in experiments on cell proliferation, invasion, and migration.

**Conclusion:**

This study provides a predictive tool for endometrial cancer. The classification based on the status of CD8+ T cells can distinguish the prognosis and treatment response, highlighting the potential of this model in personalized treatment.

## 1 Background

Endometrial cancer (EC) is the second most common malignant tumor of female reproductive system. According to the data of GLOBOCAN 2018, there were 382069 new cases and 89929 deaths in 2018 (1), and the incidence of EC is still rapidly rising in China (2). In contrast to other common cancers, the incidence and annual mortality rates of endometrial cancer are increasing (3, 4). According to the pathological characteristics, EC can be divided into two types: endometrioid endometrial cancer (EEC) and uterine serous carcinoma (USC). It is generally believed that EEC contains most EC and has a good prognosis; However, USC contained more aggressive histologies and had worse prognosis (5–7).

Most endometrial cancer can be detected by endometrial biopsy in the early stage, which greatly improves the prognosis and survival rate of patients. However, there are still a considerable number of patients who have distant metastasis when they are found, and another part of patients are not sensitive to chemotherapy, which lead to the increased risk of cancer progression or recurrence, and reduce the survival rate and quality of life of patients (8). Maybe the higher risk can be explained by inter- tumoral and intra-tumoral heterogeneity (9). Therefore, it is important to adopt updated techniques to discuss possible cell subpopulations in EC.

The tumor microenvironment (TME) refers to the cellular environment in which tumor cells exist (10). The tumor microenvironment plays a crucial role in the occurrence, maintenance, and progression of endometrial cancer (11, 12). In the normal endometrium, T cells occupy the predominant subset of immune cells, accounting for 40-80% of CD45+ immune cells (13, 14). For patients with endometrial cancer, tumor-infiltrating T cells(TIL)are also the predominant component of the endometrial cancer microenvironment. Studies have demonstrated that CD8+ T cells are essential in controlling tumor progression. They achieve this by recognizing antigens and directly killing cancer cells via perforin, granzymes, and other cytokines (15). Conversely, CD4+ T cells coordinate various immune responses, either enhancing or suppressing the immune defense against cancer cells (16).

Multiple research efforts have underscored the importance of cytotoxic CD8+ T cells in controlling tumor progression (17, 18). Pathological studies have confirmed that the presence of CD8+ tumor-infiltrating lymphocytes in endometrial cancer can predict a better prognosis (19–21). Furthermore, relevant pathological studies have also demonstrated an inverse correlation between the presence of CD8+ T cells and histological grade, myometrial invasion, and lymph node metastasis. This strongly suggests the resistance role of CD8+ T cells against tumor progression (22). Additionally, the phenomenon of lower T cell density in advanced endometrial cancer further supports the notion that tumors are more likely to progress to later stages when cytotoxic T cell infiltration decreases (23, 24). In endometrial cancer, CD8+ T cells often exhibit a state of functional impairment, which may be exacerbated by various inhibitory signals within the tumor microenvironment (16). This dysfunction could also be related to the tendency of the endometrium to favor a regulatory immune environment in its healthy state (25). Furthermore, in postmenopausal women, while the total number of CD8+ T cells increases in endometrial adenocarcinoma, their cytotoxic activity significantly decreases (26). Considering that most women are at risk of developing endometrial cancer during or after menopause (27), the risk of tumor progression is significantly increased. Additionally, the activity of CD8+ T cells may be significantly reduced due to bystander effects or interaction-induced exhaustion with malignant cells, leading to the emergence of an inhibitory tumor microenvironment (28, 29).

Simply understanding the presence of CD8+ T cells is not sufficient; it is also essential to delve deeper into their internal differentiation and gene activation status. Traditional staining methods are limited to cell identification and cannot provide information about the intracellular state. Therefore, methods that can better measure the transcriptome of specific T cell subsets should be prioritized (30). Simultaneously, this necessitates the adoption of higher-resolution techniques to study the tumor microenvironment and CD8+ T cells in endometrial cancer.

Single-cell RNA sequencing (scRNA-seq) allows for the simultaneous assessment of the full gene expression profiles of thousands of individual cells within tumor tissue. Based on characteristic genes, these cells can be classified into specific subgroups at single-cell resolution. Recent studies have evaluated prognostic factors in tumors based on single-cell features (31–33). The tumor microenvironment in endometrial cancer may play a significant role in prognosis and treatment resistance (34, 35), Single-cell technologies will help us further understand the intricacies within. As for T cell subgroups, some studies recommend assessing the presence based on transcriptomic activity (30, 36). Some molecular biomarkers used for immunotherapy have been developed and validated in clinical practice, including PD-1 or PD-L1 expression levels (37), tumor mutational burden (TMB) (38, 39) and TILs (40).

However, these methods lack certain standards in practical use and show fluctuations in predictive efficacy. The effectiveness of immunotherapy often depends on the degree of immune escape caused by infiltrating T cells expressing inhibitory receptors (41). Therefore, in this study, we aimed to elucidate the immune infiltration characteristics of CD8+ T cells in endometrial cancer. To identify CD8+ T cell-related genes with high clinical value, we integrated bulk-seq and scRNA-seq data from endometrial cancer samples. This approach aimed to elucidate the fundamental mechanisms of CD8+ T cells in tumor progression, develop new molecular stratification and prognostic features, and assist in determining the benefits of different treatment modalities for patients to achieve maximum clinical benefit.

## 2 Method

### 2.1 Data download and processing

We downloaded RNA sequencing data for 578 uterine corpora endometrial carcinoma (UCEC) cases, along with 542 mutation datasets and the patients’ Masked Copy Number Segment data from the TCGA database (https://portal.gdc.cancer.gov/). Additionally, we accessed the UCSC Xena website (http://xena.ucsc.edu/) to download clinicopathological characteristics, including age, grade, TNM stage, and MSI value, as well as clinical outcomes of the corresponding UCEC patients.

We utilized GEO datasets GSE21882 to validate the prognostic value of the immune signature, and GSE78220, PRJEB23709, and IMvigor210 to assess the immune signature’s predictive value for immunotherapy response. Datasets E-MTAB-11552, GSE120490, GSE216872, and GSE205209 were employed to explore the relationship between CD8T cells and the development of endometrial cancer.

Five primary endometrial cancer patients from GSE173682 were selected for analysis using their scRNA-seq data to investigate the role of CD8T cells in endometrial cancer.

### 2.2 Evaluation of immune cell infiltration

The distribution of immune cells in each endometrial cancer tissue sample is assessed using the CIBERSORT algorithm. Additional tumor microenvironment components, including matrix scores and tumor purity, are evaluated with the ESTIMATE package (42). To quantify the relative abundance of Tumor-Infiltrating Cells in UCEC patients, the Single-sample Gene Set Enrichment Analysis (ssGSEA) algorithm was used. This algorithm, featured in Bindea et al.’s study, provided 29 gene sets that identify various TIC types (43). Concurrently, a comprehensive evaluation of the pan-cancer tumor microenvironment immune function was conducted, incorporating 29 features. These include angiogenesis, anti-tumor cytokines, co-stimulatory ligands and receptors, various trafficking characteristics (e.g., granulocyte, myeloid, effector cell), M1 characteristics, myeloid-derived immunosuppression, MHC class I and II molecules, tumor proliferation rate, and more. It also considers interactions within the microenvironment and involves cellular components like cancer-associated fibroblasts, B cells, effector cells, endothelial cells, macrophages, dendritic cells, NK cells, T cells, and regulatory T cells (44). Additionally, T cell subpopulations were identified using ImmuCellAI, providing a detailed view of their role within the tumor microenvironment (45). Kaplan-Meier (KM) curves were plotted to explore the correlations of immune cell infiltration level with overall survival (OS) and disease-free survival (DFS).

Due to the dual nature of immune function, which can both promote and inhibit tumor progression, we utilized enrichment scores from 29 pan-cancer tumor microenvironment immune functions. Hierarchical clustering was employed to categorize the samples into two groups: one characterized by high expression of pro-inflammatory markers and the other by high expression of inhibitory markers. Within these groups, samples with enrichment scores exceeding the group mean were classified as immune-activated in the pro-inflammatory group, and conversely, those below the mean in the inhibitory group were considered immune-suppressed.

We conducted differential gene expression analysis on TCGA UCEC RNAseq data between the activated and suppressed groups (relative to tumor-adjacent tissue) using the R limma package. The selection criteria for differentially expressed genes (DEGs) were set as an absolute log fold change (logFC) greater than 2 and an adjusted P-value of less than 0.05.

### 2.3 Weighted gene coexpression network analysis

Weighted Gene Coexpression Network Analysis (WGCNA) is a systematic biological method used to explore patterns of genetic association across various samples, aiming to identify clusters of genes with high co-variation. Initially, we performed a scale-free topology analysis to determine an appropriate soft thresholding power for network construction. Using this power, we constructed an adjacency network matrix among genes.

Subsequently, we calculated the topological overlap matrix and its corresponding dissimilarity from the adjacency matrix. The dissimilarity-based clustering of the topology matrix led to the generation of different gene modules through adaptive branch pruning of hierarchical clustering dendrograms, maintaining a specified minimum number of modules.

Finally, we assessed the correlations between these gene modules and clinical traits using Pearson correlation analysis. We focused on and selected the gene module that exhibited the strongest correlation with CD8+ T cell presence, potentially linking it to key immunological behaviors in the study context.

### 2.4 Establishment of risk model for the prognosis of EC

We utilized the clinical data of patients with endometrial cancer from The Cancer Genome Atlas (TCGA) database. Univariate Cox regression analysis was applied, and resampling was conducted 1000 times within the dataset to identify genes associated with prognosis. Subsequently, Least Absolute Shrinkage and Selection Operator (LASSO) Cox analysis and multivariate Cox regression analysis were further carried out. The regularization parameter was determined through the cross-validation method. After 10 rounds of cross-validation, a curve was plotted between the logarithm of the regularization parameter λ and the partial likelihood deviation. The λ value corresponding to the minimum partial likelihood deviation was determined as the optimal regularization parameter, and the corresponding regression coefficients were extracted. Genes corresponding to non-zero regression coefficients were obtained, and these genes were used to construct a multivariate Cox proportional hazards model. The stepwise regression method was employed to optimize the model, with variables selected bidirectionally. These computations were implemented using the R package glmnet.

The formula for calculating the risk score is as follows:

Risk Score=coef1×exp1+coef2×exp2+…+coefn×expn

where “coef” represents the coefficient, “coefn” is the coefficient related to the survival of the gene, and “expn” denotes the expression level of the gene. Patients were categorized into low-risk and high-risk groups based on the median value of the risk score among endometrial cancer patients. Kaplan-Meier curve analysis, time-dependent receiver operating characteristic (ROC) curves, and the area under the curve (AUC) were used to evaluate the sensitivity and specificity of the model for survival prediction (46).

### 2.5 Predictive nomogram establision

We incorporated clinical characteristics of endometrial cancer patients, including age, stage, and grade, into the risk model for both univariate and multivariate Cox regression analysis to determine if the risk model is an independent variable.

Additionally, we utilized clinical characteristics and the risk model to construct a nomogram. The Hosmer-Lemeshow test was used to generate a curve supporting the consistency between predicted results and actual outcomes, demonstrating the predictive ability of the nomogram (47).

### 2.6 Functional analyses based on the risk model

The DEGs were first identified between 2 groups with different scores. GSEA, a method designed to assess the concerted behavior of functionally related genes forming a set, was carried out on the DEGs to reveal underlying pathways involved in tumor biology behavior using the R “ClusterProfiler” package.

### 2.7 Response to immunotherapy and chemotherapy in clusters

We utilized the maftools software package to process Mutation Annotation Format (MAF) data of endometrial cancer patients, as mutation burden and microsatellite instability are important indicators related to tumor immunity.

Furthermore, we obtained the Immune Phenotype Score (IPS) of endometrial cancer patient tumor samples from The Cancer Immunome Atlas (TCIA) (https://tcia.at/home) to infer patient response to immunotherapy by comparing these indicators between two subgroups (48, 49). We obtained scores related to immune dysfunction and exhaustion from the Cancer Immunome Dysfunction and Exclusion (TIDE) tool (http://tide.dfci.harvard.edu) (50). Using the R package Easier, we obtained scores predicting patient response to immunotherapy and compared them between two subgroups, along with scores from a series of previous studies (51). We downloaded datasets from the GEO database containing immunotherapy efficacy and survival data, computed model scores within this dataset, and compared the scores with patient response and survival. Finally, we obtained the activity intensity of different steps in immune activation in TCGA samples from the Tumor Immune Profiling (TIP) website, estimating the relationship between scores and different immune activation steps (52).

### 2.8 Anticancer drug sensitivity analysis

The Genomics of Drug Sensitivity in Cancer (GDSC) database (https://www.cancerrxgene.org/) and The Cancer Therapeutics Response Portal(CTRP) (http://portals.broadinstitute.org/ctrp/) was accessed, and using these data to calculated the predicted half maximal inhibitory concentration (IC50) values of commonly used anticancer drugs. The IC50 was compared between the two groups (53).

### 2.9 PARP inhibitor response prediction

According to the literature, genetic and epigenetic alterations o*f ARID1A, ATM, ATR, BAP1, BARD1, BLM, BRIP1, CHEK1/2, FANCA/B/C/D2/E/F/G, MRE11A, NBN, PALB2, RAD50, RAD51, RAD51B*, and *WRN* play a crucial role in the Homologous Recombination (HR) mechanism. Clinical trials have suggested that PARP inhibitors are more active in BRCA-mutated patients, followed by HR-deficient (HRD) and HR-proficient (HRP) subgroups. We then evaluated the mutation frequencies of HRD-related genes between different groups.

### 2.10 Single-cell analysis

The raw single-cell expression matrix underwent quality control filtering by removing cells with mitochondrial gene percentage > 20% and total expressed gene counts < 300, UMI < 500, and UMI > 50,000. Batch effects between samples were eliminated using the R package harmony. Dimensionality reduction was performed using the “RunUMAP” function in the R package Seurat (version 4.1.0) with parameters reduction = “harmony” and dims = 1:50. For the remaining cells, global scaling normalization method “LogNormalize” was applied, which normalizes feature expression for each cell based on total expression, multiplying by a scale factor (default is 10,000), and log-transforming the result. Principal component analysis was conducted on the top 2000 variable genes. Shared nearest neighbor modularity optimization clustering algorithm was utilized with a resolution of 1.2, using the “FindClusters” function to identify cell clusters. Non-linear dimensionality reduction using Uniform Manifold Approximation and Projection (UMAP) was employed for visualization and filtering of clustered single-cell RNA sequencing data. Differential expression genes were identified using the FindAllMarkers function in Seurat, and Wilcoxon algorithm was applied to compare differential genes between cell clusters. Marker genes were selected based on adjusted p-value (Wilcoxon rank-sum test) < 0.05 and |logFC| > 0.25 (54, 55).

Gene set enrichment analysis: The clusterProfiler R package was used to perform gene ontology (GO) and Kyoto Encyclopedia of Genes and Genomes (KEGG) enrichment analysis on the differentially expressed genes between cell clusters (56).

We utilized SCENIC to infer the gene regulatory network (GRN) of T cells. Using the human hg38 reference genome, we searched for DNA motifs within a range of 10kb upstream and downstream of the transcription start sites. This step aimed to define the scope of the GRN establishment. These algorithms predict regulatory relationships between genes based on gene motifs and gene expression data. The resulting data were standardized (z-score) across cell clusters and overlaid onto a diffusion map to reveal the GRN structure in different cell types and clusters. The proportion of significantly upregulated genes (or regulons) from a given set of genes was evaluated using AUC scores (57).

CellChat utilizes network analysis and pattern recognition methods to predict cell signaling transmission. In order to identify potential interacting cell types, the CellChat method was employed. The database was set to “ChatDB.human”, and default parameters were used (FDR-adjusted p-value < 0.05). The “computeCommunProb” function was executed to infer the probability and strength of cell-cell communication. The “mergeCellChat” function was used to combine CellChat objects for each stage (58).

Initially, a Monocle object was created using the original UMI count gene-cell matrix via the newCellDataSet function. The lowerDetectionLimit was set to 0.1, while the expressionFamily was configured to negative binomial size. Subsequently, dimensionality reduction was carried out using the reduceDimension function, employing the DDRTree method with max_components set to 2. Following dimensionality reduction, differential gene testing was conducted to identify genes exhibiting significant expression differences either temporally or across different cell states. Additionally, cytotrace was utilized to assess the differentiation hierarchy among various subtypes of CD8+T cells (59).

### 2.11 Cell culture

EC cell lines (HEC‐1A and Ishikawa) were obtained from the China Center for Type Culture Collection (CCTCC). HEC‐1A and Ishikawa cells were cultured with Dulbecco’s modified Eagle’s medium (DMEM; Gibco) supplemented with 10% (v/v) fetal bovine serum (FBS; Gibco), and 1% penicillin-streptomycin (MRC, Jintan). Cells were cultured in a thermostatic incubator at 37°C and 5% CO2. Cells were harvested for further experiments when they reached 70%–80% confluence.

Lipofectamine2000 (Invitrogen) was used to transfect the Plasmids into cells. Lentivirus containing small hairpin RNA (shRNA) targeting human *ASB2* and control shRNA lentivirus were purchased from Shanghai Genechem Co.Ltd. Cells were cultured in 6-well cell culture plates (2 × 10^5^ cells per well).Plasmids were transfected into Ishikawa while shRNA were transfected into HEC‐1A according to the manufacturer’s protocol. The effect of transfection was assessed by qPCR analysis and Western blotting.

### 2.12 Cell viability assays

HEC‐1A and Ishikawa were seeded into 96-well microplates at a density of 2000 cells per well. Additionally, 200 μL of PBS was used around the edges of the 96-well plate to prevent edge effects. After culturing the cells for 24, 48, and 72 hours, the CCK8 reagent (Vazyme) was diluted in serum-free medium at a ratio of 1:10. Care was taken to protect it from light. The mixture was then incubated in a CO2 incubator for 2 hours, after which the absorbance at 450 nm was measured using an enzyme marker.

### 2.13 Wound healing assay

Cells were seeded into a 6-well plate at a density of 1×10^5 cells per well and cultured to 90% confluence for the scratch assay. A scratch was made in the middle of each well using a 200 μl pipette tip guided by a ruler. Subsequently, each well was washed with 1 mL PBS to remove detached cells and debris, and the initial scratch width was photographed and recorded under a fluorescence inverted microscope. Following this, cells were cultured in serum-free medium, and images were captured with an Olympus microscope after 24, 48, and 72 hours of incubation. The scratch width was measured by drawing straight lines along the healing edge of the cells using PS software, and the scratch healing rate was calculated. The scratch healing rate is proportional to the cell migration ability.

### 2.14 Transwell assay

An 8µm pore size 24-well Transwell chamber (Corning) was used, coated with Matrigel matrix (Corning). 1×10^5 EC cells were seeded into the upper chamber in serum-free medium, while the lower chamber contained medium with 10% FBS as a chemoattractant. After 48 hours of incubation, cells in the lower chamber were fixed in 4% paraformaldehyde and stained with 0.1% crystal violet. Cells penetrating the lower chamber were randomly photographed and counted under a microscope.

### 2.15 RNA Isolation and qPCR

Total RNA was extracted using the RNAsimple Total RNA Kit (TIANGEN, Beijing, China) and reverse-transcribed into cDNA using the HiScript SuperMix (Vazyme). Subsequently, RT-qPCR was performed using the ChamQ Universal SYBR qPCR Master Mix (Vazyme) on a CFX96™ instrument (Bio-Rad Laboratories, Inc.). mRNA levels were calculated using the 2^−ΔCt method. The primer sequences used were as follows:

*ASB2*-F 5′-GCGACCGCTCAGAGTTACTG-3′

*ASB2*-R 5′-TCTCGCCTGTGATGACTCAG-3′

*CCL5*-F 5′-GAGTATTTCTACACCAGTGGCAAG-3′

*CCL5*-R 5′-TCCCGAACCCATTTCTTCTCT-3′

*CD3G*-F 5′-GGGATGTATCAGTGTAAAGG-3′

*CD3G*-R 5′-CAGCAATGAAGTAGACCC-3′

*BATF*-F 5′-AAAGCGAGCGACATGTCCCT-3′

*BATF*-R 5′-TTTTCTTTAAAGCATTTATT-3′

*KIAA1755*-F: 5′-ATGTCTCTCGCCGTCTCCAG-3′

*KIAA1755*-R: 5′-CGGATGCTGTTGCTATGGCC-3′

*GAPDH*-F: 5′-ACCCGCCCTATCTCAACTACC-3′

*GAPDH* -R: 5′-AGGACACCATAATGACAGCC-3′

### 2.16 Western blotting

Cells or tissues were lysed with RIPA lysis buffer (Servicebio) and mixed with 5× loading buffer, followed by boiling for approximately 10 minutes in a water bath at 100°C. Prepared protein samples were separated by 10% SDS-PAGE at constant voltage and transferred onto PVDF membranes. After blocking with 5% skim milk (Servicebio) for about 1 hour at room temperature, the membranes were incubated with primary antibodies overnight at 4°C. Subsequently, the membranes were incubated with horseradish peroxidase-conjugated secondary antibodies (Thermo Fisher Scientific, 1:10,000) for 1 hour at room temperature. After repeated washing with TBST, protein bands were detected using the ECL chemiluminescence ultra-sensitive chromogenic reagent (Vazyme). The primary antibodies used were those against *ASB2* (PA5-29476, Invitrogen, diluted 1:1500) and glyceraldehyde-3-phosphate dehydrogenase (GAPDH; 10494-1-AP, proteintech, diluted 1:20,000).

### 2.17 Statistical analysis

The Student’s t - test and F - test were used to determine the significant differences and variances between groups respectively. Pearson’s correlation analysis was used to conduct the correlation analysis. The comparison of multiple and paired non - parametric continuous variables was achieved by the Kruskal - Wallis test and the Wilcoxon test respectively. The Kaplan - Meier (KM) method was used to plot the survival curves. The bootstrap cross - validation was applied to evaluate the predictive power of various regression modeling strategies. A calibration curve was plotted in the bootstrap samples and then tested in the subjects not included in the bootstrap samples. A P - value less than 0.05 was considered statistically significant. All statistical analyses were performed using the statistical software R version 4.0.5.

## 3 Result

### 3.1 EC TME immune cells infiltration analysis

TME is pivotal in the onset and progression of cancer. We analyzed the characteristics of the tumor immune microenvironment in each sample of endometrial cancer using various methodologies.

**Figure 1:**
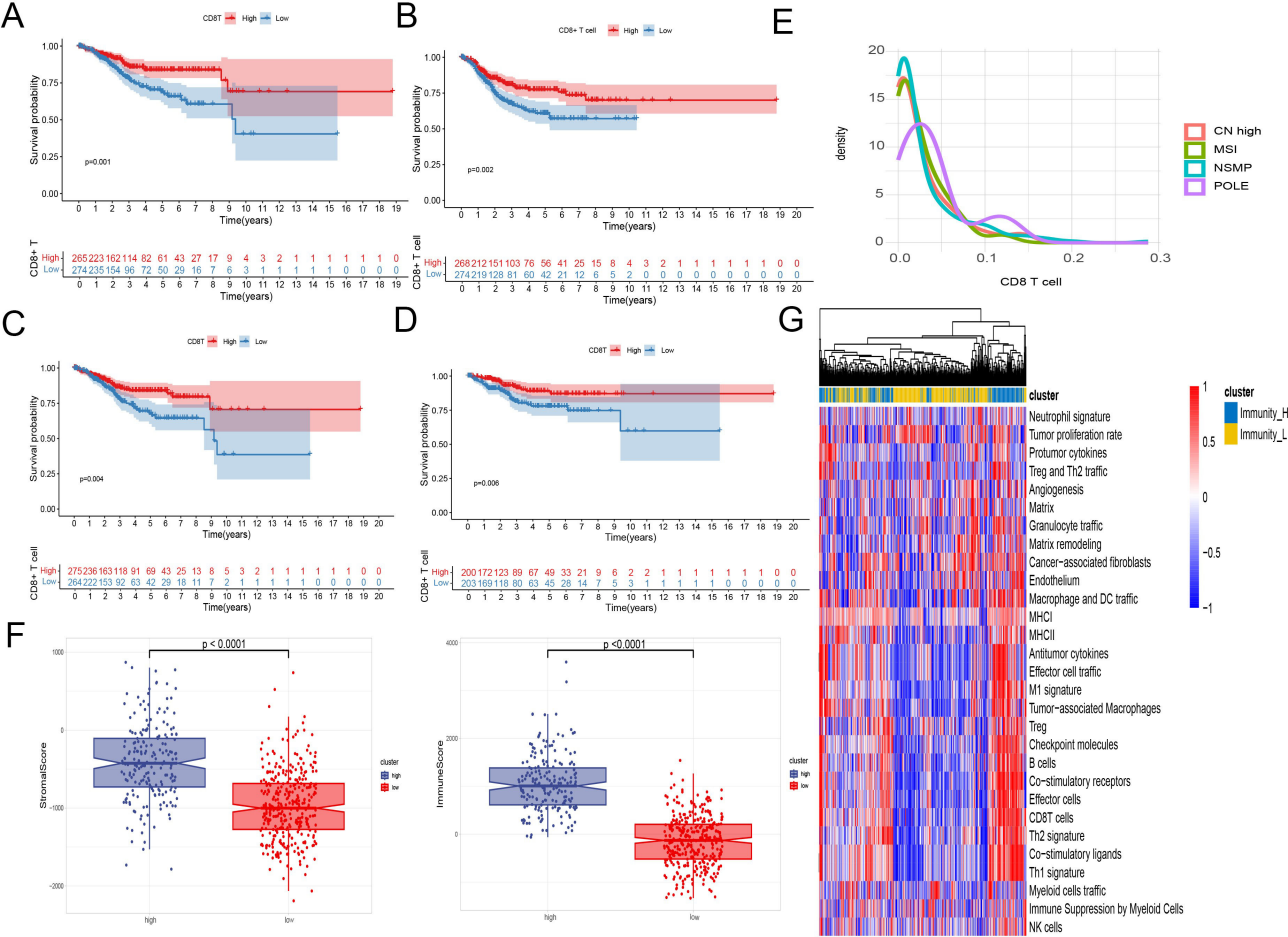
EC TME immune cells infiltration analysis. **(A–D)** Kaplan-Meier survival analysis of patient survival based on CD8+ T cell infiltration. A two-sided log-rank test is used to compare patient survival between the two groups. **(A)** The infiltration level of CD8+ T cells was calculated using the CIBERSORT algorithm, and the survival data were represented by OS. **(B)** The infiltration level of CD8+ T cells was calculated using the CIBERSORT algorithm, and the survival data were represented by PFS. **(C)** The infiltration level of CD8+ T cells was calculated using the ssGSEA algorithm, and the survival data were represented by OS. **(D)** The infiltration level of CD8+ T cells was calculated using the ImmuCellAI algorithm, and the survival data were represented by OS. **(E)** Density plot of CD8+ T cells between TCGA type. **(F)** Immune scores, stromal scores, and tumor purity between samples with high and low levels of CD8+ T cell infiltration. **(G)** A heatmap was used to display the anti - tumor and pro - tumor immune characteristics of the tumor microenvironment. The grouping was based on a comprehensive consideration of anti - tumor and pro - tumor factors.

Employing the CIBERSORT algorithm and feature genes of 29 types of cells, we estimated the distribution of immune cells in each sample. Kaplan-Meier survival analysis confirmed the anti-tumor effect of CD8+ T cell infiltration, indicating that patients with higher levels of CD8+ T cell infiltration had significantly longer OS and PFS (P < 0.05) (**Figures 1A–D**).

Interestingly, analysis across multiple GEO datasets showed that CD8+ T cell infiltration did not significantly change in metastatic and recurrent patients. However, in the GSE120490 dataset, metastatic patients exhibited significantly higher levels of CD8+ T cells compared to non-metastatic patients (**Supplementary Figure 1**).

Regarding the molecular subtypes of endometrial cancer, the density plot of CD8+ T cells from TCGA data indicated significant infiltration only in the POLE mutation group, while the other three subtypes showed no significant differences in CD8+ T cell infiltration (**Figure 1E**).

Using ESTIMATE to assess stromal and immune scores, as well as calculate tumor purity for each sample, we observed significant differences in immune scores, stromal scores, and tumor purity between samples with high and low levels of CD8+ T cell infiltration (**Figure 1F**).

We evaluated the enrichment scores of 29 descriptors of the tumor microenvironment. Through heatmap analysis, a positive correlation was found between CD8+ T cell infiltration levels and other anti-tumor immune activities, such as antitumor cytokines, co-stimulatory ligands, and co-stimulatory receptors. In contrast, factors associated with tumor progression like angiogenesis, cancer-associated fibroblasts, and the matrix showed no correlation or a negative correlation with CD8+ T cell infiltration (**Figure 1G**).

### 3.2 Identification of CD8+ T cell activation-related gene

Using enrichment scores that described 29 tumor microenvironment features, subgroups at different hierarchical levels were delineated through hierarchical clustering. These subgroups were then analyzed against the average pathway activity within each to distinguish between an immune activation group and an immune suppression group. Differential expression analysis between these groups led to the identification of 1,268 DEGs listed in the **Supplementary Material**.

**Figure 2:**
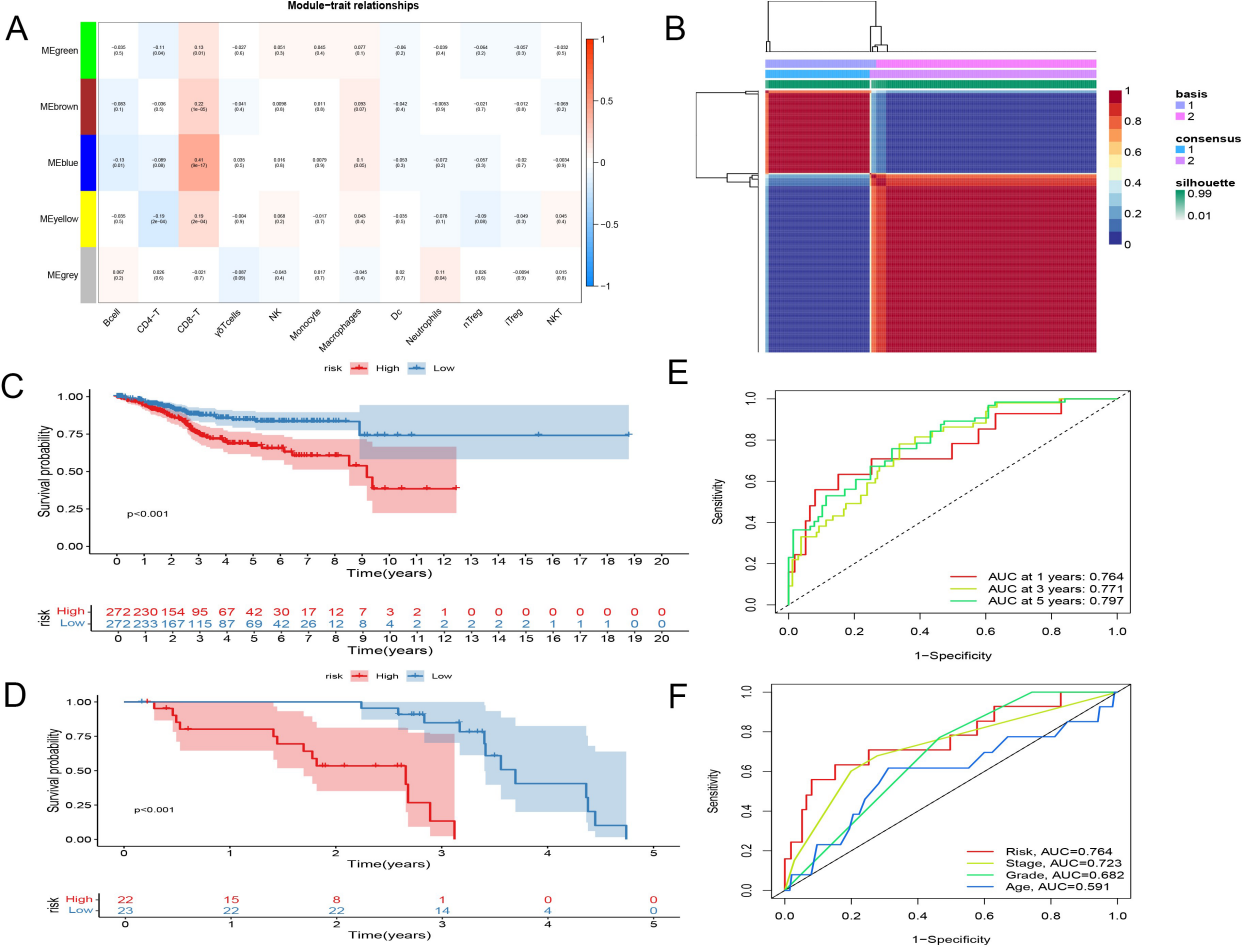
Identification and establishment of CD8+ T Cell Activation Model. **(A)** WGCNA showed blue module being the most significantly correlated with CD8+ T cells. **(B)** Consensus matrix when k = 2. The grid where rows and columns intersect represents an independent sample. Consistency is represented by the color of the matrix, from dark blue representing 1 to white representing 0. **(C, D)** Kaplan-Meier survival analysis. **(C)** entire TCGA set. **(D)** GSE21882 set. **(E)** Time-related ROC analysis of TCGA. **(F)** Time-related ROC analysis of GSE21882.

Subsequently, through the ImmuCellAI website, we determined the proportions of various T cell subtypes in endometrial cancer patients from the TCGA database. Leveraging these DEGs, key modules most correlated with CD8+ T cells in the TCGA cohort were identified. During the construction of the co-expression network, a soft thresholding power of 3 was selected, achieving a scale-free topology fitting index of 0.9. This process culminated in the formation of five modules through dynamic adaptive branch pruning, with the blue module being the most significantly correlated with CD8+ T cells (r=0.26, P<0.05) (**Figure 2A**).

### 3.3 NMF analysis of CD8+ T cell activation-related gene features

The gene features related to CD8+ T cell activation, identified earlier, were analyzed using Non-negative Matrix Factorization (NMF) clustering to explore their relationships with the clinical characteristics of endometrial cancer. At a clustering variable of (k = 2), the highest intra-group correlation was observed, suggesting that this represents the most stable classification. This finding was further validated by t-SNE analysis, which confirmed significant differences between the two groups.

Clinically, the differences between these two groups were profound, with patients in Type 1 exhibiting significantly lower overall survival and disease-free survival compared to those in Type 2. This indicates the existence of two distinct states of CD8+ T cell activation-related genes: one that promotes activation and is favorable for survival, and another that has the opposite effect (**Figure 2B**, **Supplementary Figure 2**).

### 3.4 Establishment and external validation of CD8+ T cell activation model

The LASSO Cox analysis is helpful for improving the prediction accuracy of statistical models while reducing the risk of overfitting. Therefore, we first applied univariate Cox proportional hazards regression to identify independent prognostic factors. Subsequently, a LASSO Cox analysis with ten-fold cross-validation was carried out to calculate the regression coefficients of the prognostic factors and further determine the genes to be included in the model. Finally, a multivariate Cox proportional hazards model was constructed using the included genes, and the model was optimized by the stepwise regression method. In addition to evaluating this model using a separate external dataset, we also improved the reliability of the prognostic model by repeated sampling within the group during the process of model construction. The predictive prognostic model was constructed, comprising CD8+ T cell activation-related genes—*ASB2*, *BATF*, *CD3G*, *KIAA1755*, and *CCL5*—using lasso regression analysis. The linear prediction model was established based on the weighted regression coefficients of these five prognostic-related genes:

Risk Score=(−1.5989×*ASB2*)+(−2.7822×*BATF*)+(−0.5837×*CD3G*)+(−1.2417×*KIAA1755*)+(0.9590×*CCL5*).

In this model, *CCL5* was the only gene showing a significant positive correlation with the risk score, whereas *ASB2, BATF, CD3G*, and *KIAA1755* exhibited significant negative correlations with the risk coefficient.

To confirm the stability and reliability of our model, we used the TCGA dataset as the internal training set and GSE21882 as the external validation set. Patients were divided into low-risk and high-risk groups based on the median value of the risk score. The predictive ability of the model was validated using Kaplan-Meier survival analysis across the EEC set, SEC set, and the entire TCGA dataset (**Figures 2C, D**, **Supplementary Figure 2**). Results showed significantly longer OS in the low-risk group compared to the high-risk group. Time-related ROC analysis confirmed the consistent prognostic accuracy of the risk score, demonstrating the model’s robust predictive performance (**Figures 2E, F**).

### 3.5 Validation of the CD8+ T cell activation model as an independent prognostic indicator and nomogram construction

**Figure 3:**
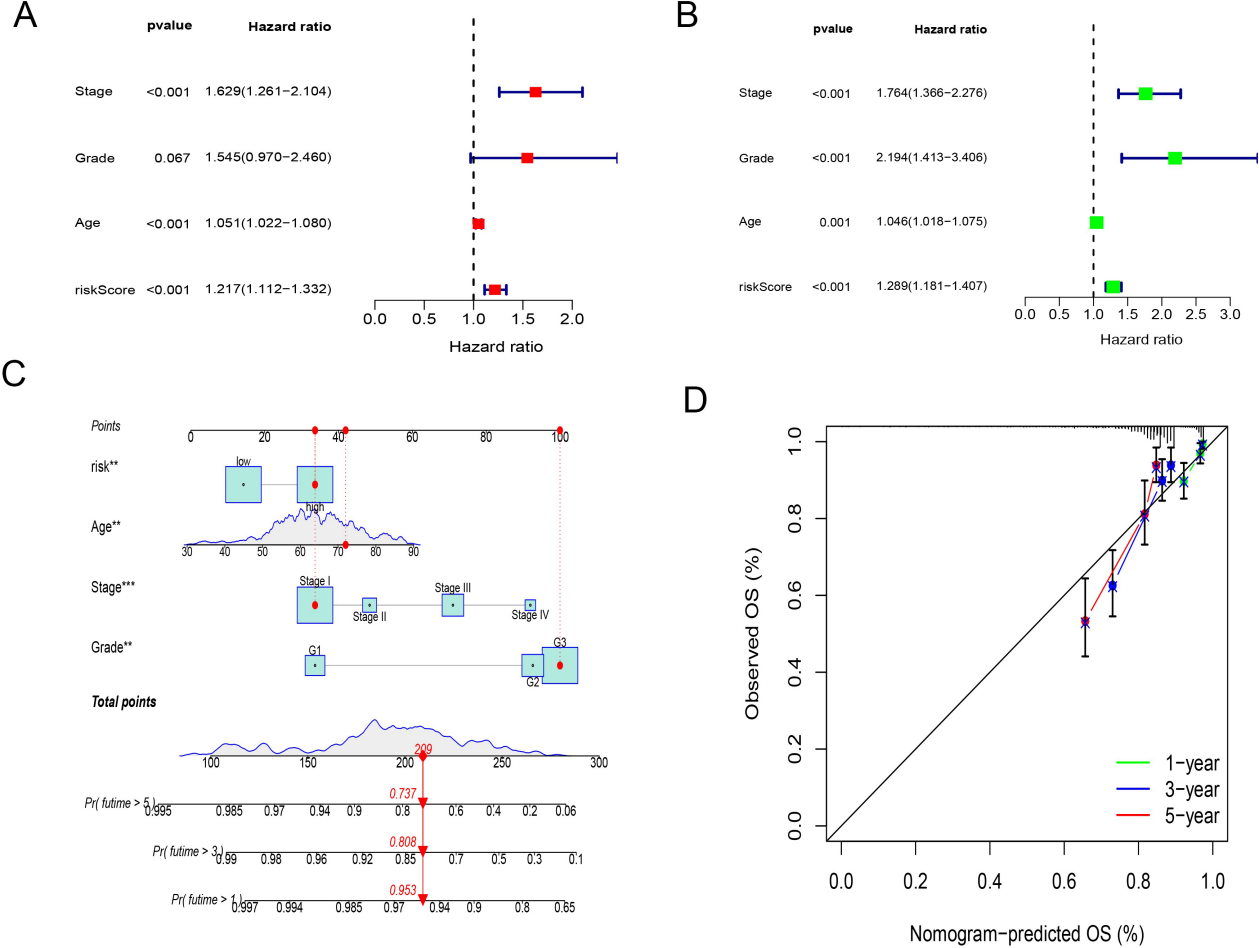
Validation of the CD8+ T cell activation model as an independent prognostic indicator and nomogram construction. **(A)** univariate Cox regression analyses of CD8+ T cell activation model. **(B)** multivariate Cox regression analyses of CD8+ T cell activation model. **(C)** A nomogram shows the score correspondence table. The predictive factors include age, disease stage, tumor grade, and risk score. **(D)** Calibration curves for 1-year,3-year and 5-year overall survival.

In this study, 548 endometrial cancer patients with clinical indicators such as age, tumor staging, and grading were analyzed. We conducted univariate and multivariate Cox regression analyses to assess whether the CD8+ T cell activation model could serve as an independent prognostic factor. The hazard ratio (HR) was 1.289 with a 95% confidence interval (CI) of 1.181-1.407 in the univariate Cox regression analysis, and an HR of 1.217 with a 95% CI of 1.112-1.331 in the multivariate Cox regression analysis, both with p-values < 0.001. These results indicate that the CD8+ T cell activation model is independent of clinical features such as age, tumor staging, and grading, and qualifies as an independent prognostic indicator (**Figures 3A, B**). We also evaluated the independence of the risk model using GEO dataset analyses, which similarly demonstrated that the risk coefficient is independent of pathological staging and tumor grading (**Supplementary Figure 3**).

A nomogram was successfully constructed that incorporates risk scores and clinical features, including age, tumor grading, and staging (**Figure 3C**). Calibration curves for 3-year and 5-year OS showed high consistency between the predicted mortality rates and actual mortality rates, underscoring the nomogram’s excellent predictive ability (**Figure 3D**).

### 3.6 Multi-omics features of the CD8+ T cell activation model

**Figure 4:**
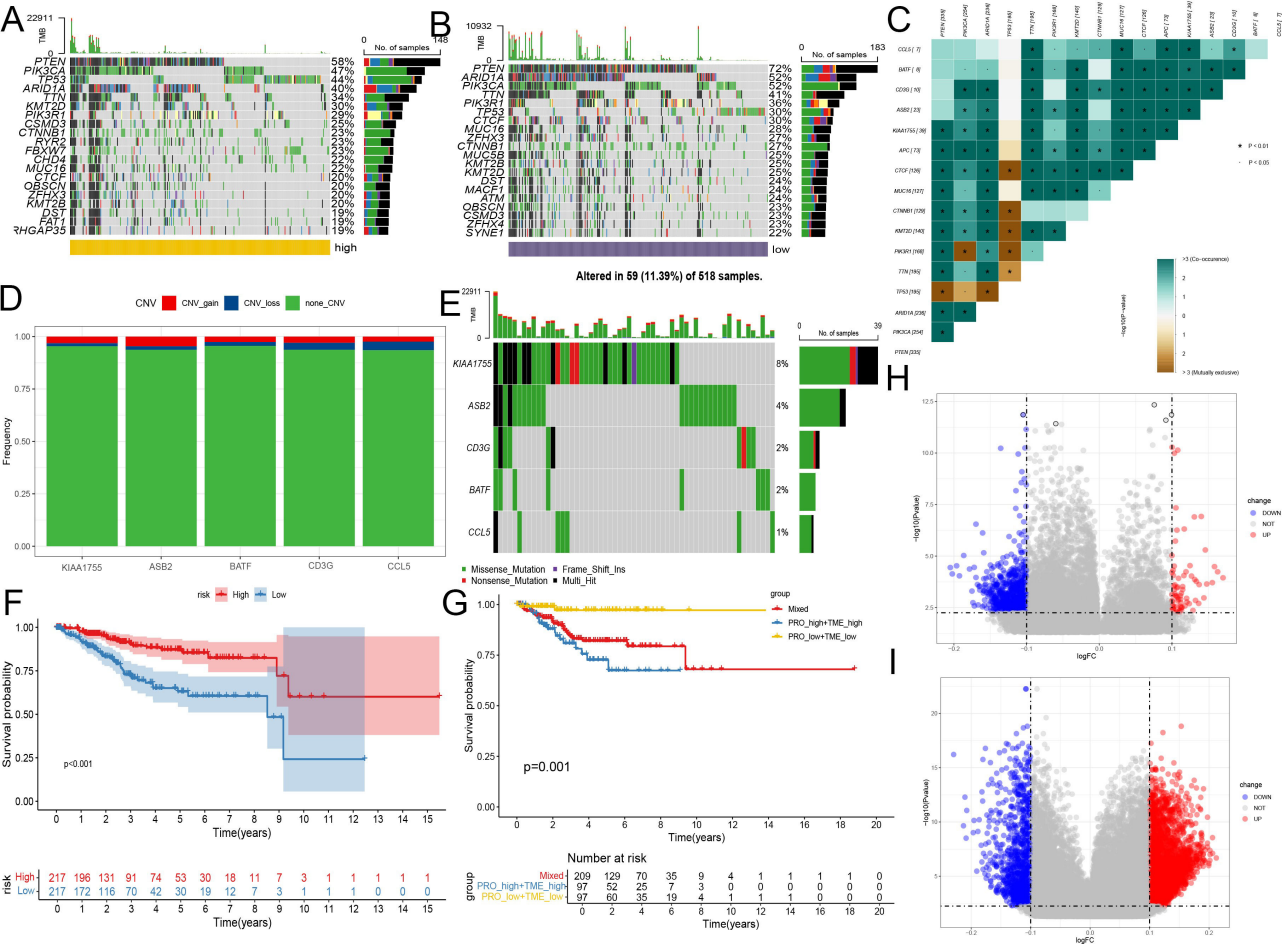
Multi-omics features of the CD8+ T cell activation model. **(A, B)** Genomic changes associated with CD8+ T cell activation model in EC. **(A)** Waterfall Plot displays the top 20 driver genes with the highest mutation frequencies in high groups. **(B)** Waterfall Plot displays the top 20 driver genes with the highest mutation frequencies in low groups. **(C)** Heatmap shows the collinearity analysis of model gene mutations with PTEN, ARID1A, and PIK3CA. Green represents the co - occurrence of mutations, and dark yellow represents mutual exclusivity. **(D)** Bar chart shows the changes in gene copy numbers in the CD8+ T cell activation model. **(E)** Waterfall Plot displays the changes in mutations of genes from the CD8+ T cell activation model. **(F)** Kaplan-Meier analysis of patient survival based on protein prediction model, the levels of ERALPHA, PAX8, P16INK4A, and PR were obtained from the TCGA database. **(G)** Kaplan-Meier analysis of patient survival for combining protein model and the CD8+ T cell activation model. **(H)** Volcano plot showed differences in methylation between the high and low CD8+ T cell groups. **(I)** Volcano plot showed differences in methylation between the high and low-risk groups of the CD8+ T cell activation mode. *, P < 0.01, proving that the correlation between the two mutations is reliable.

Using the TCGA database, we visualized and analyzed somatic mutation data for high-risk and low-risk groups using the maftools package. The visualization displays the top 20 driver genes with the highest mutation frequencies in high and low subgroups. We identified five genes showing discrepancies between the two groups, with differences in mutation probabilities for the same genes, indicating significant divergence in tumor mechanisms between the subtypes. The three genes with the highest mutation frequencies in the high-risk (HR) group were *PTEN* (58%), *TP53* (44%), and *PIK3CA* (47%). In the low-risk (LR) group, the most frequently mutated genes were *PTEN* (72%), *ARID1A* (52%), and *PIK3CA* (52%). This also explains the concentrated appearance of SEC in the HR group (**Figures 4A, B**).

Further analysis of the mutations in genes included in our model revealed that, except for *KIAA1755*, the mutation probabilities of other genes were relatively low, suggesting their differential expression might primarily occur at transcriptional and post-transcriptional stages. Co-linearity analysis confirmed positive correlations between mutations in model genes and mutations in *PTEN, ARID1A*, and *PIK3CA*, but these were not significantly associated with *TP53* mutations. These findings indicate that model genes are relatively stable in endometrial cancers driven by *TP53* mutations (**Figure 4C**).

Using the GISTIC 2.0 data from UCEC, we demonstrated the changes in gene mutations in the CD8+T cell activation model. *KIAA1755* and *ASB2* are the two most frequently mutated genes, but their mutation rates are only 8% and 4%, respectively. Similar to mutations, copy number increases or decreases are also rare, indicating that the chromosomes of these genes are relatively stable (**Figures 4D, E**).

*ER, PR, PAX8*, and *P16* expression are commonly used in the pathological examination of endometrial cancer to guide treatment selection. We examined whether the combined use of the CD8+ T cell activation model with the expression of these proteins could better predict patient survival. Proteomics studies confirmed that all three proteins were associated with prognosis. Consequently, we employed LASSO Cox analysis to construct a protein prediction model using the TCGA database, combining it with the CD8+ T cell activation model. We found that survival outcomes for the high-risk group in both the protein model and the RNA high-risk score group were significantly worse than the other three groups, while the survival of the low-risk group in both models was the best, with no significant differences observed between the other two groups. This indicates that the combined use of both models can more effectively predict patient prognosis (**Figures 4F, G**).

Lastly, based on methylation data from TCGA, we analyzed the differences in methylated sites between the high and low CD8+ T cell groups, as well as between the high and low-risk groups of the CD8+ T cell activation model. The results showed that differences in methylation were more significant between the two groups of the CD8+ T cell activation model, indicating that the model can better reflect the heterogeneity among endometrial cancer patients and distinguish the origins of transcriptional pre-regulation that may influence differences in immune activity (**Figures 4H, I**).

### 3.7 Function enrichment analysis of the CD8+ T cell activation model

To further delineate the functional disparities between the two subgroups of endometrial cancer patients, we employed Gene Set Variation Analysis (GSVA) to identify genes that were upregulated and downregulated between the groups. This analysis was pivotal in uncovering the underlying biological processes differentiating the subgroups.

**Figure 5:**
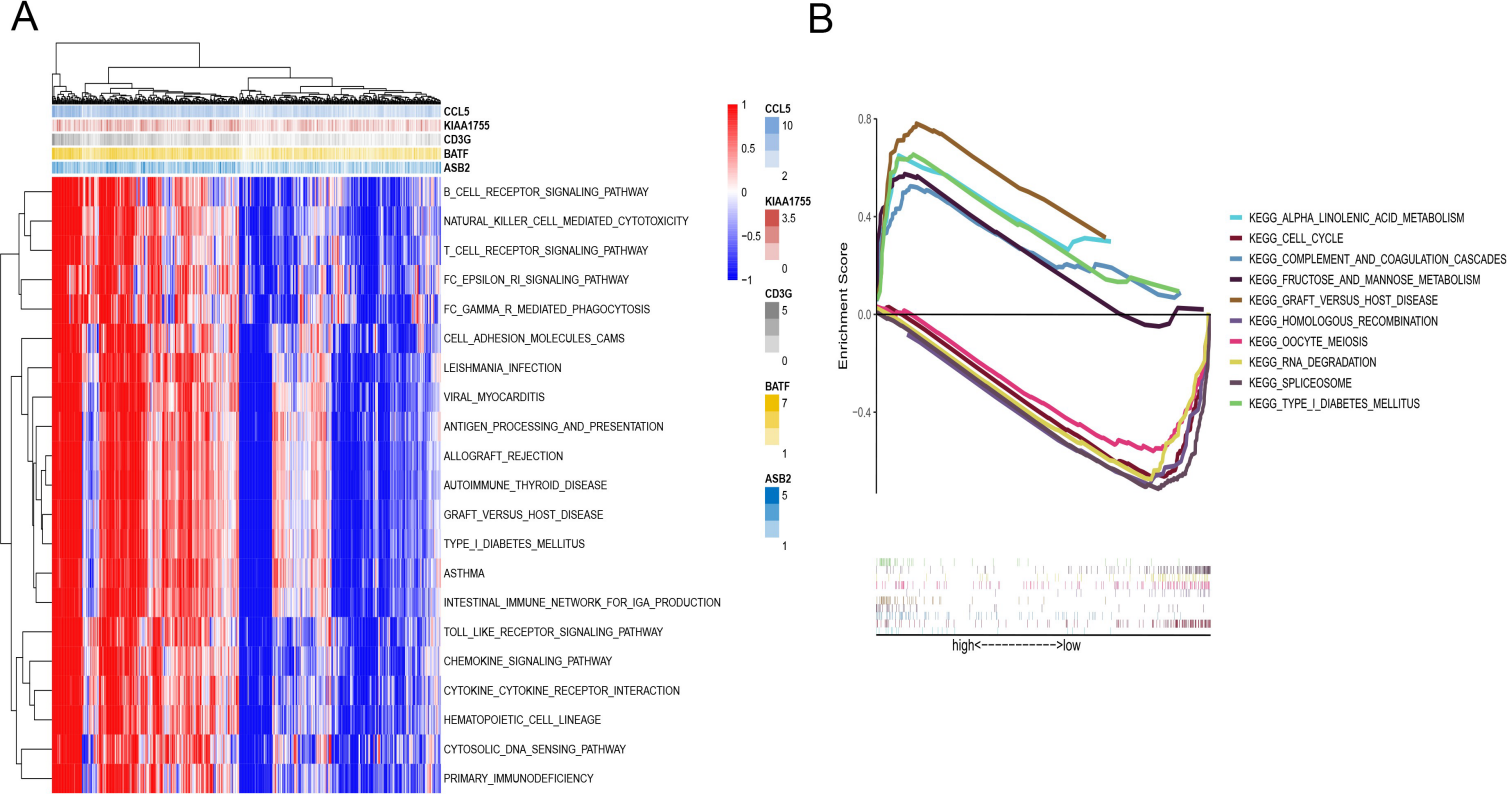
Function enrichment analysis of the CD8+ T cell activation model. **(A)** GSVA of immune pathway analysis between HR and LR. **(B)** GSEA analysis between HR and LR.

Following GSVA, we conducted immune pathway analysis to gain insights into the affected biochemical pathways. The results indicated that the model genes are strongly linked to several critical signaling pathways, including cell adhesion molecules, chemokine signaling pathways, B cell receptor signaling pathways, and T cell receptor signaling pathways (**Figure 5A**). These pathways are integral to the modulation of immune responses and cellular interactions within the tumor microenvironment, highlighting their potential role in tumor progression and patient prognosis.

Further analysis revealed distinct pathway enrichments between the two risk groups. Samples from the high-risk group showed significant enrichment in pathways involved in alpha-linolenic acid metabolism and the cell cycle. These pathways are often associated with rapid tumor growth and aggressive cancer behavior. In contrast, pathways related to spliceosomes and RNA degradation were more actively engaged in the low-risk group (**Figure 5B**).

### 3.8 Predicting and validating the efficacy of immunotherapy

**Figure 6:**
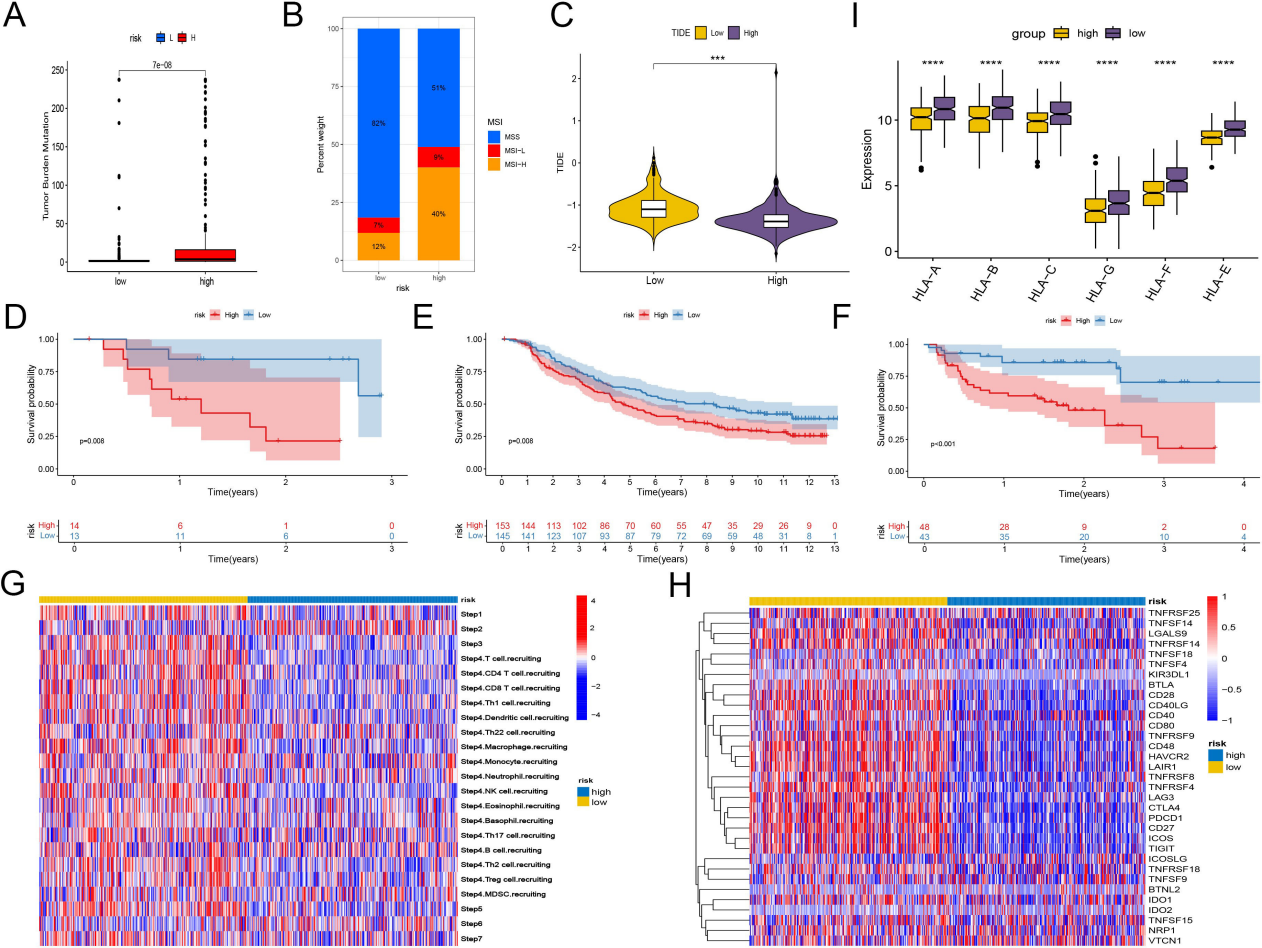
Predicting and validating the efficacy of immunotherapy. **(A)** Box plot of TMB between high-risk group and low-risk group. **(B)** Bar plot of MSI between high-risk group and low-risk group. **(C)** Box plot of TIDE score between high-risk group and low-risk group. **(D–F)** Kaplan-Meier analysis of patient survival based on CD8+ T cell activation model from three patient cohorts undergoing immunotherapy. **(D)** GSE78200. **(E)** imv210. **(F)** PRJEB23709. **(G)** Heatmap shows the differences in TIP scores between the high and low groups. The TIP score represents a series of cell recruitment steps. **(H)** Heatmap displays the differences in the expression of immune checkpoints between the high and low groups. **(I)** Box plot illustrates the differences in the expression of MHC class I molecules between the high and low groups. ***, P < 0.001; ****, P < 0.0001, vs High risk group.

Immune checkpoint blockade (ICB) therapy, a critical category of immunotherapy, operates by inhibiting signals that suppress T-cell activation, thereby facilitating tumor-reactive T-cells to effectively combat tumors (60).In-depth analyses were performed to explore differences in the immune microenvironment among subtypes of the CD8+ T-cell activation model. We found that both TMB and Microsatellite Instability (MSI) were elevated in the high-risk group compared to the low-risk group, suggesting a greater likelihood of response to immunotherapy in the high-risk group (**Figures 6A, B**).

Using the TCIA, we calculated IPS to gauge the responsiveness of endometrial cancer patients to immunotherapy. Despite similar levels of PD-L1 and CTLA4 positivity, the low-risk group had higher IPS, indicating potential sensitivity to immune checkpoint therapy. And another scoring method TIDE also proves that the low-risk group has a higher probability of responding (**Figure 6C**, **Supplementary Figure 4**).

To further validate the utility of risk scoring in predicting immunotherapy outcomes, three patient cohorts undergoing immunotherapy were analyzed. Consistently, the high-risk group showed lower survival rates, while the low-risk group had higher probabilities of clinical remission and complete remission, underscoring a correlation between lower risk scores and favorable responses to immunotherapy (**Figures 6D–F**, **Supplementary Figure 4**).

In addition, we compared the immunological activities among different groups using the TIP score. The low-risk group showed increased activities related to the recruitment of NK cells and CD8+ T cells, as well as enhanced functions of the chemokine system, indicating a greater potential in mobilizing tumor-infiltrating immune cells. The evaluation of CD8+ T cell infiltration among different groups revealed a significant increase in CD8+ T cell infiltration in the low-risk group, strongly confirming the enhanced recruitment (**Figure 6G**, **Supplementary Figure 4**). However, further studies are needed to assess the possible differences in tissue-resident immune cells among different groups.

The expression analysis of immune checkpoints and major histocompatibility complex (MHC) class I molecules revealed significant differences: Although the high-risk group generally had higher expression levels of various immune checkpoints, the expression of MHC class I molecules was lower, suggesting a changed immune landscape. In contrast, the low-risk group had higher expression levels of identified immune checkpoint targets, such as *PDCD1, (TIGIT), HAVCR2, ICOS*, and *CTLA4*, implying that there might be both immune activation and immune exhaustion in this group (**Figures 6H, I**). Scoring the T cell cytotoxicity and IFN-γ in the high-risk and low-risk groups, the results showed that the scores in the low-risk group increased, supporting the enhanced anti-tumor effect and the occurrence of immune activation in the low-risk group (**Supplementary Figure 5**). By deconvoluting the TCGA data according to the annotated single-cell data of EC, it was found that the tissue-resident memory (TRM) cells in the low-risk group increased significantly (**Supplementary Figure 5**). Meanwhile, within the single-cell data, the TRM cells had the lowest risk scores. Considering that TRM cells mainly play an anti-tumor role in EC (61, 62), this may partly explain why the low-risk score group has a better prognosis.

### 3.9 Evaluation of the CD8+ T cell activation model for chemotherapy sensitivity and DNA repair pathway mutations

Chemotherapy remains a pivotal treatment for endometrial cancer. To ascertain the clinical utility of the CD8+ T cell activation model in guiding chemotherapy, we leveraged data from GDSC and CTRP. Using the Oncopredict algorithm, we calculated the half-maximal inhibitory concentration (IC50) of common chemotherapy drugs for each patient sample, which helped predict their sensitivity to these drugs.

**Figure 7:**
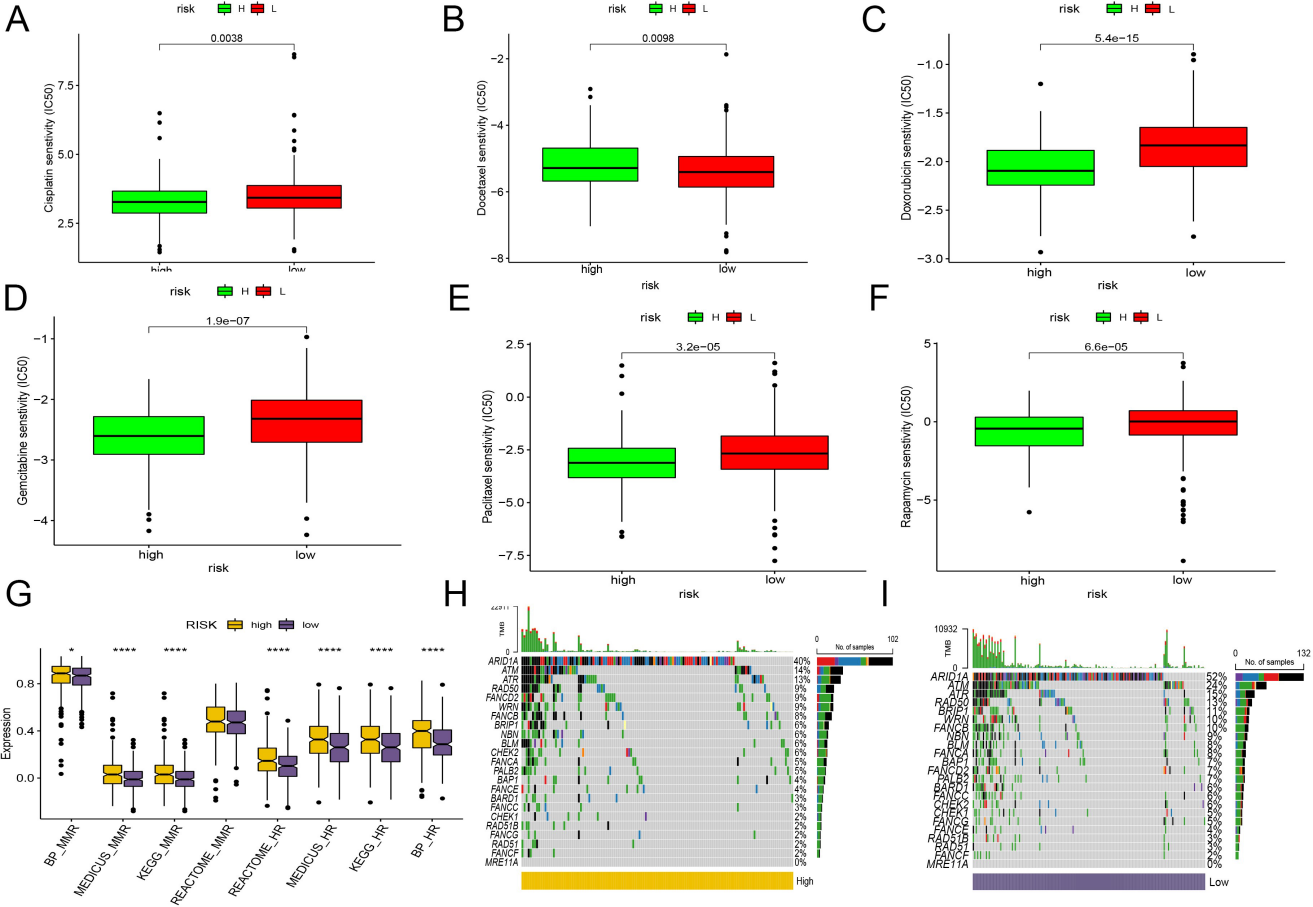
Evaluation of the CD8+ T cell activation model for chemotherapy sensitivity and DNA repair pathway mutations. **(A–F)** IC50 of common chemotherapy drugs for each patient sample, using box plot compare the sensitivity between two groups. **(A)** Cisplatin. **(B)** Docetaxel. **(C)** Doxorubicin. **(D)** Gemcitabine. **(E)** Paclitaxel. **(F)** Rapamycin. **(G)** The bar plot demonstrates the KEGG pathway enrichment of HR and mismatch repair pathways between HR and LR. **(H, I)** Genomic changes associated with CD8+ T cell activation model in EC. **(H)** mutations in genes associated with HR repair in the high-risk group. **(I)** mutations in genes associated with HR repair in the low-risk group. *, P < 0.05; ****, P < 0.0001, vs High risk group.

The analyses revealed that patients in the low-risk group generally exhibited greater sensitivity to most common chemotherapy drugs, with the notable exception of docetaxel, compared to those in the high-risk group (**Figures 7A–F**).

Further supporting personalized treatment strategies, research indicates that tumors with *BRCA1/2* mutations or those characterized by homologous recombination deficiency may benefit more from treatment with PARP inhibitors (63).

In our study, we extensively analyzed the activation and mutation status of the homologous recombination (HR) and mismatch repair (MMR) pathways, crucial for DNA repair mechanisms, across various risk groups in endometrial cancer. We found significant activation of both HR and MMR pathways in the high-risk group (**Figure 7G**). Additionally, we detected frequent mutations in genes linked to HR repair, such as *ARAD1A, ATR*, and *ATM*, in both risk groups. Notably, the gene *FANCD2* showed a higher mutation frequency in the high-risk group, while *BRIP1* was more commonly mutated in the low-risk group (**Figures 7H, I**). Despite these differences, the overall mutation spectra between the two groups remained quite similar. Interestingly, the low-risk group, characterized by a greater number of mutations and less active homologous recombination pathways, appears more likely to benefit from PARP inhibitors clinically. However, it’s important to consider that drugs targeting various pathways may still provide effective treatment options for both groups. The varied response to different therapeutic agents underscores the complexity of endometrial cancer and highlights the necessity of a personalized approach in the treatment regimen.

### 3.10 scRNA-seq analysis of endometrial cancer reveals CD8+ T cell subtypes and their functional states

In our study, we analyzed 10x scRNA-seq data from five endometrial cancer samples retrieved from the GSE173682 dataset. We initially focused on the top 2000 highly variable genes, which were visualized in the analysis. Dimensionality reduction through UMAP analysis enabled the identification of 31 distinct cell subtypes within these samples.

**Figure 8:**
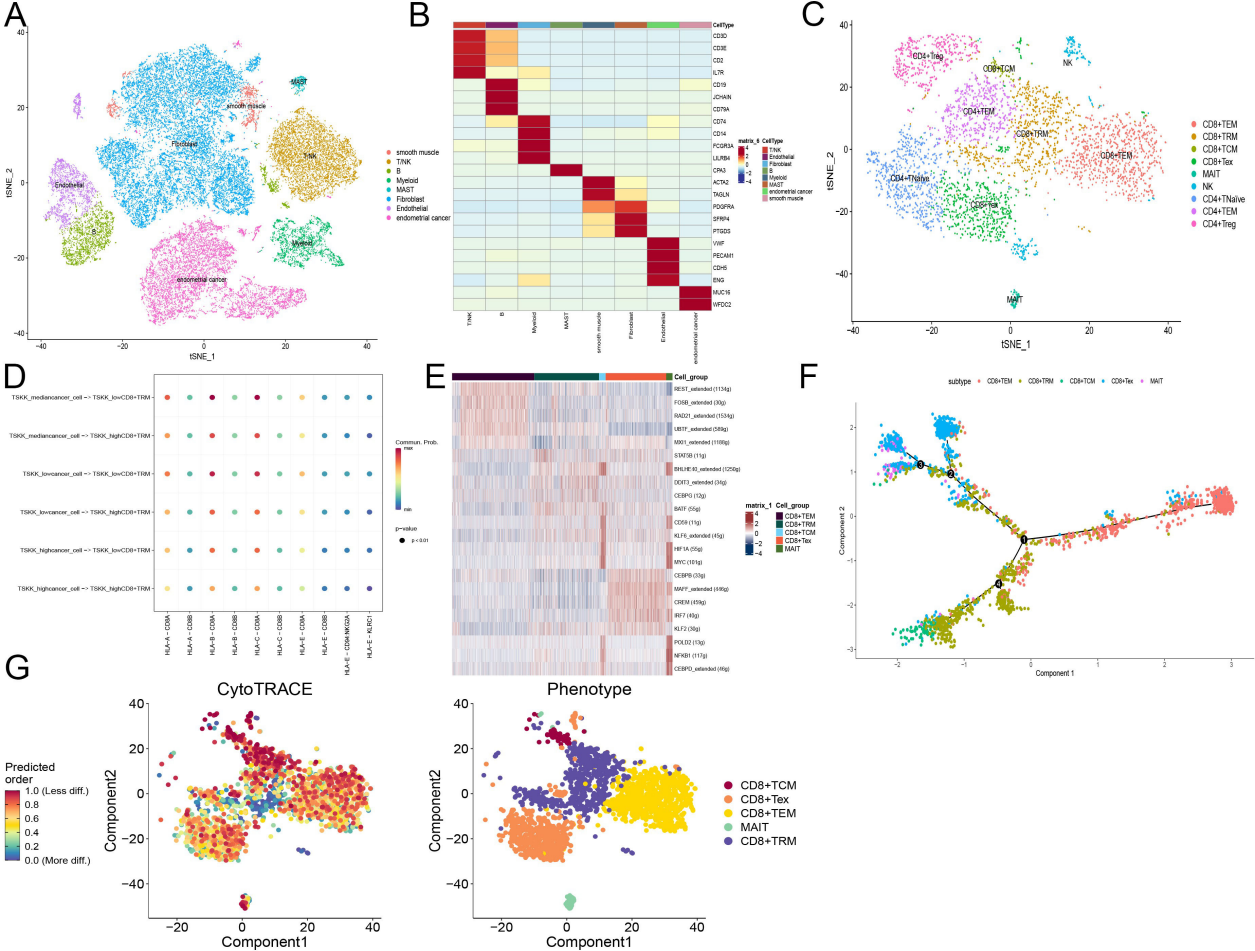
scRNA-seq analysis of endometrial cancer reveals CD8+ T cell subtypes and their functional states. **(A)** Tsne plot of cells from GSE173682 including five primary EC cases, colored by eight major cell types. **(B)** Heatmap of the top marker genes in each major cell types. **(C)** Tsne plot of T cells from GSE173682 including five primary EC cases, colored by nine major cell types. **(D)** Cellchat shows the signal intensity of MHC-I related molecules between tumor cells and tissue-resident memory T cells (TRM). Tumor cells and TRM calculate the risk coefficients according to the risk model. **(E)** Scenic calculates the activity of transcription factors in different CD8+ T cell subsets. Different colors represent different CD8+ T cell subsets. **(F)** Monocle simulates the developmental trajectory of CD8+ T cells. Different colors represent different CD8+ T cell subsets. **(G)** Cytotrace calculates the cellular stemness of CD8+ T cells. Different colors represent different CD8+ T cell subsets.

Further annotation and visual clustering of these dimensionality-reduced cell types using known cell subtype molecular markers allowed us to discern 8 major cell types, including myeloid cells, endothelial cells, smooth muscle cells, epithelial cells, B cells, MAST cells, stromal cells, as well as T and NK cells (**Figures 8A, B**).

**Figure 9:**
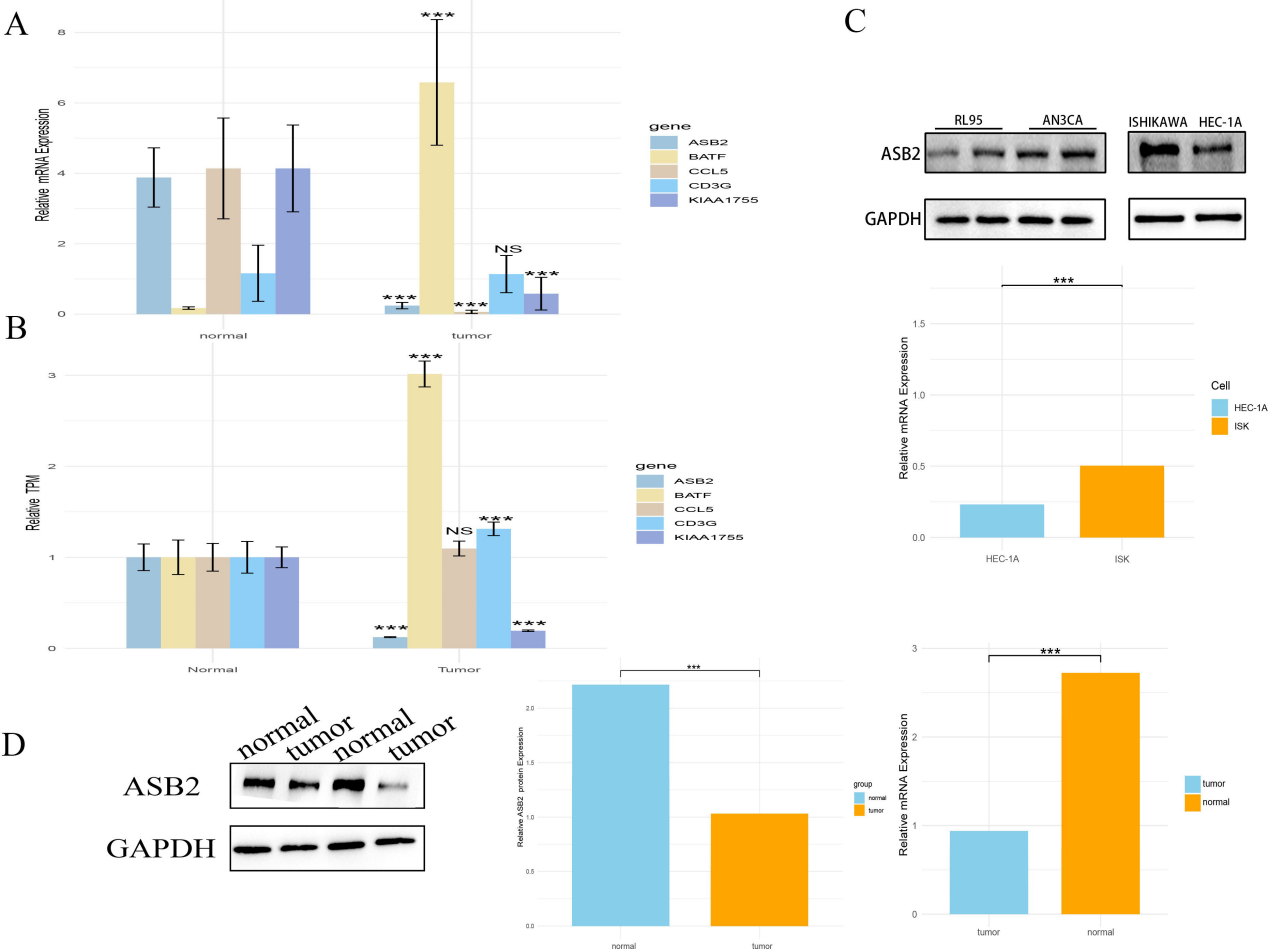
The expression of model genes in endometrial cancer cells and patient tissues. **(A)** Expression differences of the model gene between tumor and normal tissues in the TCGA database (***, P < 0.001 vs. normal). **(B)** RT-qPCR validation of model gene expression differences between tumor tissues and adjacent normal tissues in patients(***, P < 0.001 vs. normal). **(C)** RT-qPCR and WB validation of ASB2 expression in endometrial cancer cell lines Ishikawa and HEC-1A (***, P < 0.001 vs. HEC-1A). **(D)** WB validation of ASB2 expression in tumor and adjacent normal tissues in patients (***, P < 0.001 vs. normal). NS: nonsense.

For T lymphocytes, detailed clustering revealed subsets such as CD8+ and CD4+ T cells. We further characterized the states of these T cells:

- CD8+ T cells were subdivided into:
- CD8+TEM (T effector memory): Expressing *GZMA, GZMB, GZMH, GZMK*, and *NKG7*.
- CD8+TRM (T resident memory): Marked by *ITGAE, CCL4*, and *XCL1.*
- CD8+TCM (T central memory): Characterized by *CD28* and *IL7R*.
- CD8+Tex (T exhausted): Identified by expression of *CTLA4, PDCD1*, and *LAG3*.
- MAIT (Mucosal-associated invariant T): Defined by *ZBTB16* and *KLRB1*.
- CD4+ T cells were categorized into:
- CD4+TNaïve: Positive for *TCF7, IL7R*, and *CCR7*.
- CD4+TEM: Expressing *CTLA4, PDCD1*, and *HAVCR2*.
- CD4+Treg (T regulatory): Defined by *FOXP3* and *TNFRSF4* (**Figure 9C**).

Additionally, we inferred cell-cell communication networks, identifying that the MHC-I signaling pathway played a crucial role, particularly in relation to T cell interactions (**Figure 8D**). The presence of MHC-I is vital for antigen presentation to T lymphocytes, with *HLA-B* and *HLA-C* showing strong activity. Notably, the activity of these molecules was enhanced in T cells corresponding to lower CD8+ T cell activation model scores, suggesting a direct influence of these scores on the tumor-killing function of CD8+ T cells.

Transcription factor activity was analyzed using SCENIC in different CD8+ T cell subgroups:

TEM and Tex subgroups showed consistent activity in certain transcription factors, with R*EST, FOSB, RAD21, UBTF*, and *MXI1* activated in the TEM subgroup, and *CEBPB, MAFF, CREM, IRF7*, and *KLF2* in the Tex subgroup. Heterogeneity within the TRM subgroup suggested diverse origins from different precursor cells post-activation. Interestingly, TCM and MAIT subgroups displayed similar activation patterns, hinting at a deeper relationship between these cells (**Figure 8E**).

Finally, pseudo-temporal trajectories calculated using Monocle2 demonstrated progression of CD8+ T cells from TEM to both TRM and Tex subtypes, indicating that effector cells do not solely progress towards exhaustion but maintain substantial anti-tumor effects, aligning with contemporary research insights. This comprehensive analysis underscores the complexity of immune responses within endometrial cancer and highlights the importance of detailed cellular profiling in understanding tumor-immune dynamics (64).

Using Cytotrace, we calculated the stemness across different cellular subtypes within the CD8+ T cell population in endometrial cancer. The T central memory (TCM) subtype demonstrated lower stemness compared to other groups, indicative of its limited proliferation capacity due to its terminally differentiated state. This aspect was further validated by Monocle2 trajectory analysis, which confirmed the progression of CD8+ T cells from T effector memory (TEM) to both T resident memory (TRM) and T exhausted (Tex) subtypes in early-stage endometrial cancer (**Figures 8F, G**).

In terms of gene expression: *BATF, CD3G, KIAA1755*, and *CCL5* are predominantly expressed in activated T cell subtypes, suggesting their crucial roles in T cell activation and function.

*ASB2*, on the other hand, does not show significant differences in expression among different immune cells, yet interestingly, its expression appears elevated in tumor cells from patients with a higher proportion of activated T cells.

### 3.11 The expression of model genes in endometrial cancer cells and patient tissues

This study employed bioinformatics methods to analyze the expression of model genes in cancer (UCEC). The results indicate that apart from BATF, which is highly expressed in UCEC, the expression levels of *ASB2* and *KIAA1755* in tumors are relatively low, while the remaining genes did not show significant differences (**Figure 9A**). To validate these bioinformatics findings, we further examined the mRNA levels of these genes in endometrial cancer tissues and adjacent non-cancerous tissues using real-time quantitative PCR (RT-qPCR). The results were consistent with the bioinformatics analysis, confirming that the mRNA levels of *BATF* are significantly higher in the cancer tissues compared to the adjacent tissues, while the expressions of *CCL5, ASB2*, and *KIAA1755* are lower, and *CD3G* did not show significant differences (**Figure 9B**). Additionally, we measured the expression of *ASB2* in the EC cell lines Ishikawa and HEC-1A using RT-qPCR. The results demonstrated that *ASB2* expression is significantly higher in the HEC-1A cell line compared to Ishikawa. Protein testing in cell lines also validated the RT-qPCR results, with higher *ASB2* protein expression in the HEC-1A cell line compared to Ishikawa (**Figure 9C**).

Furthermore, we studied the protein level expression of *ASB2*, finding that the protein content of *ASB2* is lower in cancer tissues compared to adjacent non-cancerous tissues (**Figure 9D**).

### 3.12 Knocking down ASB2 promoted cancer cells proliferation, migration, and EMT

Based on the findings that ASB2 is underexpressed in EC tissues and cells, we hypothesized that the reduction of ASB2 might play a significant role in the progression of EC. Considering that *ASB2* is expressed at the highest level in HEC-1A cells and at a lower level in Ishikawa cells, our study chose to knock down the *ASB2* in HEC-1A cells and to overexpress it in Ishikawa cells.

**Figure 10:**
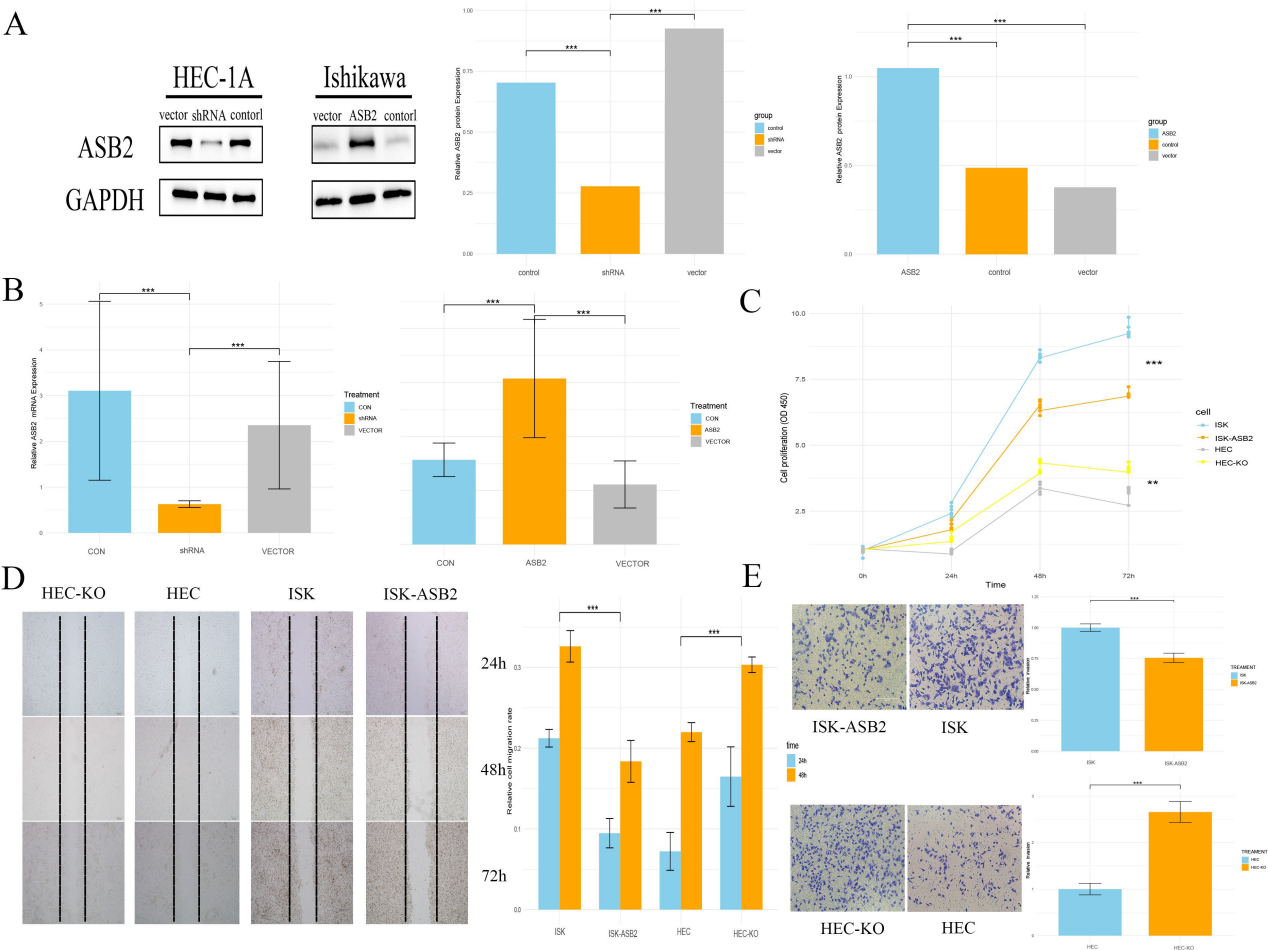
Knocking down *ASB2* promoted cancer cells proliferation, migration, and EMT. **(A)** WB validation of *ASB2* knockdown in HEC-1A cell line and *ASB2* overexpression in Ishikawa cell line (***P < 0.001 shRNA vs. control, ***P < 0.001 shRNA vs. vector, ***P < 0.001 ASB2 vs. control, ***P < 0.001 *ASB2* vs. vector). **(B)** RT-qPCR validation of *ASB2* knockdown in HEC-1A cell line and *ASB2* overexpression in Ishikawa cell line (***P < 0.001 shRNA vs. control, ***P < 0.001 shRNA vs. vector, ***P < 0.001 ASB2 vs. control, ***P < 0.001 *ASB2* vs. vector). **(C)** CCK8 assay shows that *ASB2* knockdown in HEC-1A cell line enhances proliferation, while *ASB2* overexpression in Ishikawa cell line reduces proliferation (***P < 0.001 HEC-ASB2 vs. HEC, ***P < 0.001 ISK-KO vs. ISK). **(D)** Wound healing assay shows that *ASB2* knockdown in HEC-1A cell line enhances migration, while *ASB2* overexpression in Ishikawa cell line reduces migration (Scale bar: 200 μm; ***P < 0.001 HEC-ASB2 vs. HEC, ***P < 0.001 ISK-KO vs. ISK). **(E)** Transwell assay shows that *ASB2* knockdown in HEC-1A cell line enhances invasion, while *ASB2* overexpression in Ishikawa cell line reduces invasion (Scale bar: 100 μm; ***P < 0.001 HEC-ASB2 vs. HEC, ***P < 0.001 ISK-KO vs. ISK).

Using liposome-based transfection and Lentivirus infection, we created HEC-1A cell lines with knocked-down *ASB2* and Ishikawa cell lines with overexpressed *ASB2*. The efficacy of the knockdown and overexpression was validated through RT-qPCR and WB (**Figure 10A**).

A CCK-8 assay demonstrated that silencing *ASB2* significantly reduced the proliferative ability of HEC-1A cells, while the overexpression construct showed the opposite effect, indicating that *ASB2* expression can significantly inhibit cancer cell proliferation. We conducted scratch healing and Transwell invasion assays related to the cell migration phenotype. Results from the scratch healing assays showed that cells in the overexpression group had lower healing and migration capabilities compared to the control group, while the knockdown group significantly enhanced cell migration and invasion (**Figures 10B–E**).

## 4 Discussion

The tumor microenvironment is a complex system composed of cancer-associated fibroblasts, endothelial cells, various immune cells, extracellular matrix, and cytokines. It has a significant impact on tumor progression, leading to the emergence of drug resistance and metastasis. Immunotherapy targeting immune escape caused by the tumor microenvironment, such as immune checkpoint blockade therapy, is one of the new strategies for treating endometrial cancer. Numerous clinical trials are currently underway to investigate the benefits of various immunotherapies in endometrial cancer, aiming to address the challenges posed by the tumor microenvironment and enhance treatment outcomes (65). Traditional tumor staging does not reflect the changes in the tumor microenvironment and immunity that are crucial for assessing the efficacy of immunotherapy. The demand for predicting the sensitivity to immunotherapy in EC necessitates the exploration of updated molecular markers (66). As one of the primary factors contributing to anti-tumor activity within the tumor microenvironment, CD8+ T cells have come into focus. CD8+ T cells exert direct cytotoxic effects on cell surfaces through “ligand-receptor” interactions, and they participate in cellular immunity against tumor cells by producing cytokines, tumor necrosis factor, and interferon-alpha. However, continuous exposure of T cells to antigens and/or inflammatory signals within cancer can lead to T cell exhaustion, wherein T cells lose their ability to eliminate tumors (67, 68). The interaction between PD-1/PD-L1 can exert a potent inhibitory effect on the function of CD8+ T cells, including their cytotoxicity capabilities (69). In endometrial cancer, PD-1 expression is observed in 61% of cases among TILs, and PD-L1 expression is detected in 80% of cases. Additionally, 100% of metastatic endometrial cancer cases express PD1 (70). It appears that the phenomenon of T cell exhaustion due to the action of the PD-1/PD-L1 axis is quite common in patients with endometrial cancer. Additionally, according to previous research, there is a decrease in the proportion of cytotoxic CD8+ T cells in endometrial cancer compared to normal endometrial tissue (71). This suggests that, compared to a larger number of CD8+ T cells, maintaining an activated immune state of CD8+ T cells may play a more significant role in preventing tumor progression. Therefore, we performed ssGSEA scoring on 29 immune pathways in the tumor microenvironment based on the obtained responses (44). Subsequently, through unsupervised clustering, we divided them into two groups: strong and weak immune activity, and compared the differential genes between the two groups to identify the differential immune activation genes in EC. Next, we conducted WGCNA to identify key gene clusters closely associated with EC CD8+ T cell infiltration. Subsequently, using Lasso Cox regression, we selected 5 immune-related prognostic genes. These scores not only have good prognostic predictive value with AUC > 0.6, but also stratify into two subgroups: a high-risk group with poorer prognosis associated with hypertension and more pregnancies, and a low-risk group with better prognosis, younger age, and lower clinical stage and grade. Regarding molecular subtypes of endometrial cancer, patients in the low-risk group are mainly composed of CN-L and MSI types.

EC, being a hypermutated cancer, has a higher probability of MSI, which makes it highly promising for the application of ICIs (72, 73). We aim to validate the predictive system’s value in immunotherapy by comparing the scores with features strongly associated with ICI responsiveness, including TMB, PD-L1/PD-1 expression levels, and leukocyte infiltration (74). In endometrial cancer, the POLEmut and MMRd subtypes exhibit higher PD-L1 expression levels (75). This suggests that these subtypes may be particularly responsive to immunotherapy, further emphasizing the potential utility of immune checkpoint inhibitors in the treatment of endometrial cancer.

The elevated expression of *PDCD1* in the high-risk group in our study further validates its predictive value. Furthermore, in endometrial cancer, other immune checkpoint molecules should also receive more attention. *CTLA-4* blockade has been shown to improve prognosis in endometrial cancer patients (76). Additionally, molecules such as *VISTA, TIM-3*, and *LAG3* are expressed in endometrial cancer and may serve as markers for T cell exhaustion (77–79). These molecules also exhibit differential expression, with higher expression observed in the low-risk group. As another predictive biomarker for immunotherapy, TMB is also a positive prognostic factor, negatively correlated with the score (80). Additionally, MSI has been utilized for immunotherapy prediction (81). Considering that MSI tumors often have higher levels of CD3+ and CD8+ T cells (82), the two groups based on differential activation of CD8+ T cells also exhibit differences in MSI status. Tumor neoantigens more accurately predict outcomes in melanoma patients treated with first-line anti-PD-1 or anti-CTLA-4 therapy compared to other biomarkers such as PD-L1 levels and mutational burden (50). We observed different mutational landscapes in the low-risk group, which may generate more tumor neoantigens compared to the high-risk group, thereby activating cytotoxic CD8+ T cells. MHC class I molecules presenting tumor antigens are necessary for the anti-tumor function of CD8+ T cells (83, 84). However, loss of MHC class I molecules can occur in up to 42% of endometrial cancer patients (85), potentially leading to a further decrease in the proportion of cytotoxic CD8+ T cells (86). Therefore, this study also explored the relationship between MHC class I molecules and predictive models and stratification. The results revealed that MHC class I molecules are less active in the high-risk group, potentially contributing to the decreased cytotoxicity of CD8+ T cells.

Based on existing research (74), an analysis of the composition of immune subtypes in the two types revealed that LR is primarily composed of wound healing and IFN-γ dominant subtypes, while HR includes wound healing, IFN-γ dominant, lymphocyte depleted, and inflammatory subtypes. Interestingly, within the LR immune response-active subtypes, particularly the inflammatory subtype, the majority of deceased endometrial cancer patients were concentrated. This contradicts the viewpoint in some literature that the inflammatory subtype achieving immune balance has a better prognosis. To further validate the potential of risk scoring in predicting the outcomes of immunotherapy, we selected three patient cohorts undergoing immunotherapy. The results across the three datasets were consistent, indicating that patients in the high-risk scoring group had lower survival rates and significant differences, while those in the low-risk group had higher probabilities of clinical remission and complete remission. This suggests that patients who respond to immunotherapy tend to have lower risk scores. This difference reflects the advantage of the model proposed in this study compared with the traditional pan-cancer model, as it can better reflect the anti-tumor ability of CD8 T cells in EC, rather than simply focusing on the situation of infiltration. Although macroscopically, the high-risk group has lower expression of immune checkpoint molecules, this is not reflected in the Tex cells. The high-risk group has a proportion of Tex cells similar to that of the low-risk group. In EC, lower infiltration of CD8+ T cells implies a higher possibility of non-response to immunotherapy (87).

This difference reflects the advantage of the model proposed in this study compared with the traditional pan-cancer model, as it can better reflect the anti-tumor ability of CD8 T cells in EC, rather than simply focusing on the situation of infiltration. Although macroscopically, the high-risk group has lower expression of immune checkpoint molecules, this is not reflected in the Tex cells. The high-risk group has a proportion of Tex cells similar to that of the low-risk group. In EC, lower infiltration of CD8+ T cells implies a higher possibility of non-response to immunotherapy. Although it is not possible to conclude that high-risk patients are less suitable for immunotherapy without using data from endometrial cancer patient cohorts, the differences in survival outcomes remind us that high-risk patients may have unique immune response mechanisms, and traditional immunotherapy might fail during disease progression.

ER (estrogen receptor) and PR (progesterone receptor) also play a role in the tumor immune microenvironment, which may be why combining proteomics and transcriptomics can improve prediction accuracy. Elevated levels of E2 (estradiol) in patients with endometrial cancer may directly impact CD8+ T cells and suppress their cytotoxic abilities (88). However, at the same time, the expression of ER and PR in epithelial cells brings these cells closer to CD8+ T cells, implying that they are more likely to be influenced by the anti-tumor effects of CD8+ T cells (89, 90). This suggests that common pathological markers may have broader utility in the future, but their regulatory role in the immune response of endometrial cancer remains to be further elucidated.

Chemotherapy-induced apoptosis may activate CD8+ T lymphocytes through various pathways, including depleting immune response inhibitory cells and inducing the emergence of tumor-specific CD8+ T lymphocytes (91–94). This has also been confirmed in gynecologic tumors, where CD8+ T cell infiltration remains stable even after receiving neoadjuvant platinum-based chemotherapy in ovarian cancer (95, 96). Although there is currently a lack of evidence specifically for EC, for those considered to have an immunogenic or “hot” phenotype of EC, there is a relatively greater likelihood of increased anti-tumor response following chemotherapy. Consequently, we also investigated the commonly used chemotherapy regimens in EC patients to discuss the possibility of combining chemotherapy with immunotherapy. Through the GDSC database, we validated the commonly used chemotherapy regimens in EC patients. The results revealed that low-risk group patients were more sensitive to paclitaxel, docetaxel, cisplatin, doxorubicin, gemcitabine, and rapamycin. This suggests a potential guiding role of this classification in the chemotherapy and immunotherapy of patients. Chemotherapeutic drugs used in endometrial cancer often achieve efficacy by increasing DNA damage in cancer cells. Comparisons between groups indicate that in high-risk patients, homologous recombination and mismatch repair processes are more active, suggesting that we can adopt corresponding strategies targeting this pathway.

Analysis of the dynamic immune microenvironment changes during the tumorigenic process of EEC showed tendencies of decreased proportion of cytotoxic and naive CD8+ lymphocyte population and increased proportion of CD4 Treg population, indicating immune escape during endometrial tumorigenesis (71). Our study observed that CD8+ T cells, which continue to exert their effects in early-stage endometrial cancer patients, still constitute the majority. Simply attributing this to a highly immunosuppressive microenvironment seems too hasty; further discussion is warranted on the transcriptional activation and evolutionary trends of CD8+ T cells. This study is the first to explore the prognostic significance and immune activation roles of *BATF, CD3G, KIAA1755* and *CCL5* in endometrial cancer. Although previous studies have found that the expression level of ASB2 in EC is lower than that in normal tissues, which implies that upregulating the expression of ASB2 is a potential therapeutic strategy for endometrial cancer (97). Through experiments, it discussed for the first time the expression and anti-cancer effects of ASB2 in endometrial cancer. Previous research has concentrated on the role of *ASB2* in immune cells in forming and maintaining cell scaffolding and immature morphology but lacked studies on its potential role in tumors. As the expression of *ASB2* significantly decreases during the progression from normal tissue to endometrial cancer, it is difficult to attribute the drastic decrease solely to the consumption of immune cells. The study preliminarily confirmed the anti-cancer effect of *ASB2* in endometrial cancer cell lines through experiments and suggested its correlation with being a protective prognostic factor. Additionally, although some studies have indicated a negative correlation between the presence of CD8+ T cells and clinical pathological features such as histological grade, muscular layer infiltration, and lymph node metastasis (18, 22). However, in this study, the infiltration status of CD8+ T cells obtained through transcriptomic data using methods such as CIBERSORT showed no significant relationship with recurrence, metastasis, etc. On the other hand, *ASB2*, as identified in the corresponding transcriptomic data, could play a role in distinguishing and indicating the occurrence of recurrence and metastasis. This suggests that in transcriptomic data, *ASB2* can replace complex methods, making transcriptomic data reflect potential prognosis of patients more simply and clearly. Multivariable Cox regression analysis demonstrated that this model is an independent risk factor for overall survival (OS), and ROC curves also showed that this model outperforms traditional methods such as clinical stage and grade in predicting risk. Additionally, the forest plots based on risk coefficients for predicting 3-year and 5-year OS closely matched the observed values, indicating the great potential of this new predictive system in guiding risk stratification and clinical decisions.

However, this study has some limitations. Firstly, the model was constructed using only TCGA UCEC dataset, validated using a single independent GEO dataset and a small-scale clinical sample, without validation from larger datasets. The stability of the predictive model and forest plots also requires confirmation through large-scale clinical trials. Moreover, there are similar issues in predicting immunotherapy sensitivity, as validation was only performed using data from other tumors, with limited reference value for endometrial cancer. Therefore, we plan to further collect clinical specimens and conduct more basic experiments to elucidate their specific roles, while simultaneously paying attention to the emergence of possible new external data for model validation.

## 5 Conclusion

In summary, our study provides potential predictive tools for endometrial cancer patients and contributes to further exploring the mechanisms of interaction between CD8+ T cell activation genes in endometrial cancer. Endometrial cancer can be divided into two distinct groups based on the activation status of CD8+ T cells, which have markedly different prognoses and tumor microenvironments. This suggests the potential of CD8+ T cell activation in personalized therapy. Thus, CD8+ T cell activation can serve as an important basis for prognosis prediction and treatment decisions in endometrial cancer.

## Data Availability

The original contributions presented in the study are included in the article/**Supplementary Material**. Further inquiries can be directed to the corresponding author.

## Glossary

UCEC: Uterine Corpus Endometrial Carcinoma
EC: Endometrial Cancer
EEC: Endometrioid Endometrial Cancer
USC: Uterine Serous Carcinoma
TCGA: The Cancer Genome Atlas
GEO: Gene Expression Omnibus
PRJEB: Project European Nucleotide Archive (ENA) Browser
GSEA: Gene Set Enrichment Analysis
IMvigor: (Refers to a clinical study, not an abbreviation)
RNA-seq: RNA Sequencing
scRNA-seq: Single-cell RNA Sequencing
NMF: Non-negative Matrix Factorization
t-SNE: t-distributed Stochastic Neighbor Embedding
UMAP: Uniform Manifold Approximation and Projection
WGCNA: Weighted Gene Co-expression Network Analysis
LASSO: Least Absolute Shrinkage and Selection Operator
ROC: Receiver Operating Characteristic
AUC: Area Under the Curve
HR: Hazard Ratio
CI: Confidence Interval
DEG: Differentially Expressed Gene
logFC: Log Fold Change
CIBERSORT: (A method, not an abbreviation)
ESTIMATE: Estimation of Stromal and Immune cells in Malignant Tumor tissues using Expression data
ssGSEA: Single-sample Gene Set Enrichment Analysis
KM: Kaplan-Meier
OS: Overall Survival
DFS: Disease-Free Survival
TME: Tumor Microenvironment
MHC: Major Histocompatibility Complex
NK: Natural Killer
PD-1/PD-L1: Programmed Death-1/Programmed Death-Ligand 1
MSI: Microsatellite Instability
TMB: Tumor Mutational Burden
TIDE: Tumor Immune Dysfunction and Exclusion
IC50: Half Maximal Inhibitory Concentration
GDSC: Genomics of Drug Sensitivity in Cancer
CTRP: Cancer Therapeutics Response Portal
PARP: Poly ADP Ribose Polymerase
HRD/HRP: Homologous Recombination Deficient/Proficient
UMI: Unique Molecular Identifier
TCIA: The Cancer Imaging Archive
IPS: Immune Phenotype Scores
TIP: Tumor Immune Phenotype
ICB: Immune Checkpoint Blockade
MAIT: Mucosal-Associated Invariant T cells
EMT: Epithelial-Mesenchymal Transition
RT-qPCR: Real-Time Quantitative Polymerase Chain Reaction
WB: Western Blot
CCK-8: Cell Counting Kit-8
ICIs: Immune Checkpoint Inhibitors
TILs: Tumor-Infiltrating Lymphocytes
CN-L: Copy Number Low
POLEmut: Polymerase Epsilon Mutant
MMRd: Mismatch Repair Deficient
CTLA-4: Cytotoxic T-Lymphocyte-Associated Protein 4
VISTA: V-domain Ig Suppressor of T cell Activation
TIM-3: T cell Immunoglobulin and Mucin-domain containing-3
LAG3: Lymphocyte Activation Gene-3
IFN-γ: Interferon Gamma
ER: Estrogen Receptor
PR: Progesterone Receptor
E2: Estradiol
