# Supplementary material for: CD8+ T cell activation in endometrial cancer: prognostic implications and potential for personalized therapy
Source: Front Immunol. 2025 Apr 28;16:1542669. doi: 10.3389/fimmu.2025.1542669 (PMC12066579; doi:10.3389/fimmu.2025.1542669)
Supplement: Supplementary file 1 [file DataSheet1.pdf]

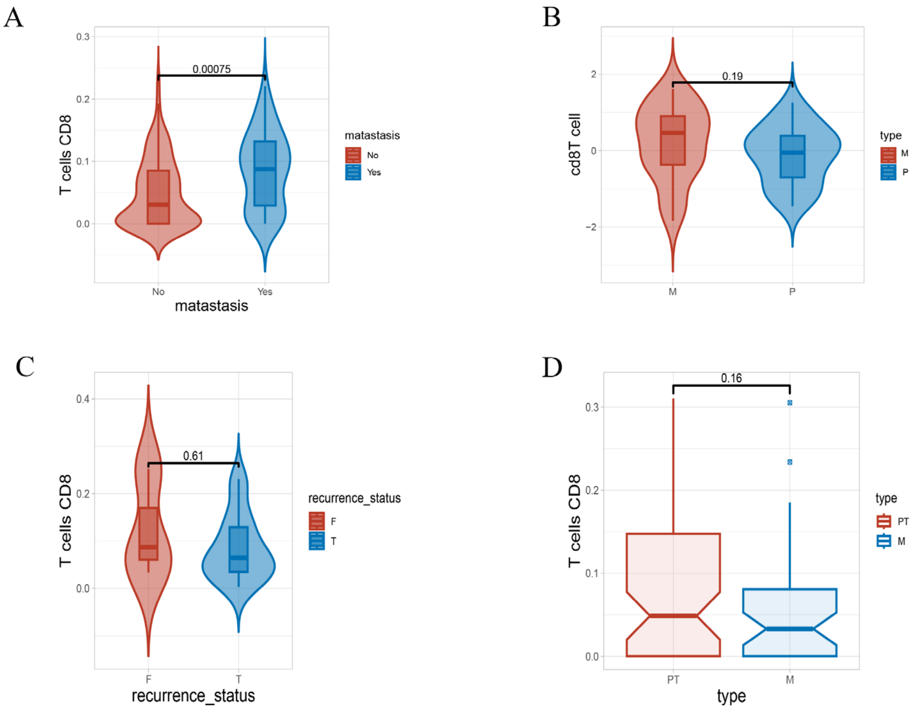

Supplementary Fig. 1

The infiltration situation of CD8+ T cells in EC at different stages

(A - B) Metastasis (C) Recurrence (D) Metastasis

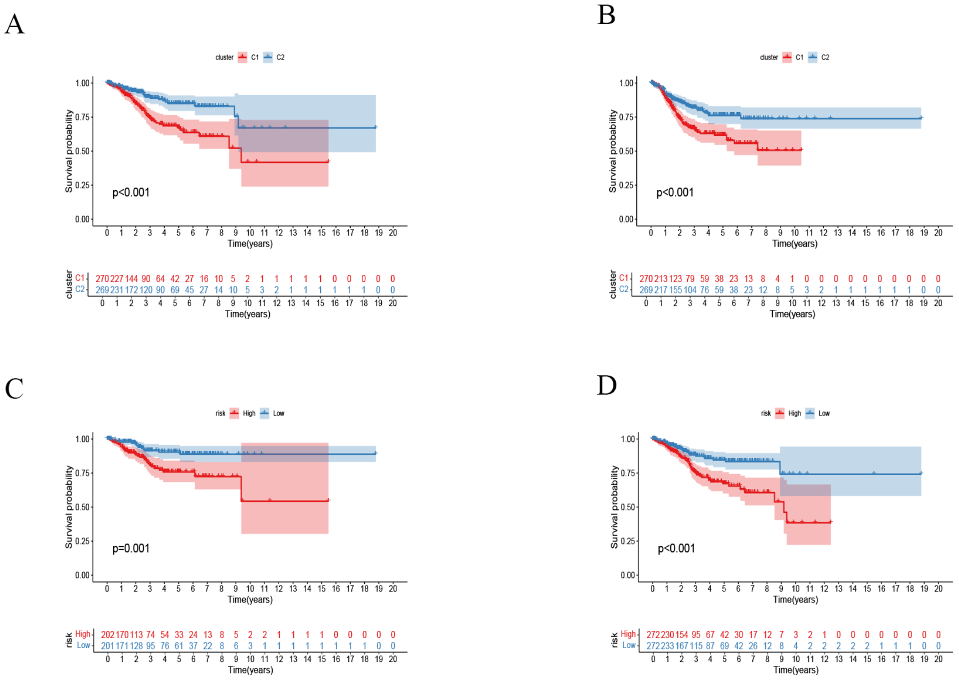

Supplementary Fig. 2

(A) Kaplan-Meier analysis of patient survival based on NMF subtypes , and the survival data were represented by OS

(B) Kaplan-Meier analysis of patient survival based on NMF subtypes , and the survival data were represented by PFS

Kaplan-Meier analysis of patient survival based on CD8+ T cell activation model from TCGA

(C) EEC set

(D) SEC set

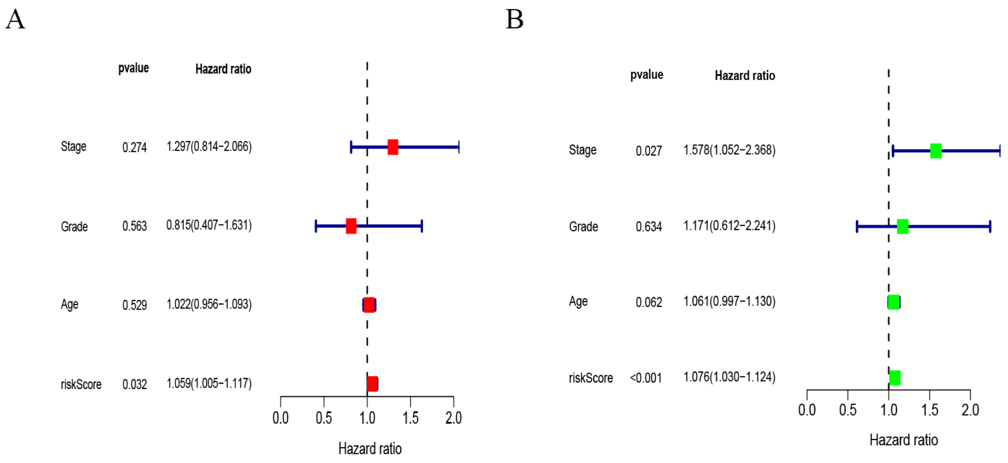

Supplementary Fig. 3

(A) univariate Cox regression analyses of CD8+ T cell activation model from GEO

(B) multivariate Cox regression analyses of CD8+ T cell activation model from GEO

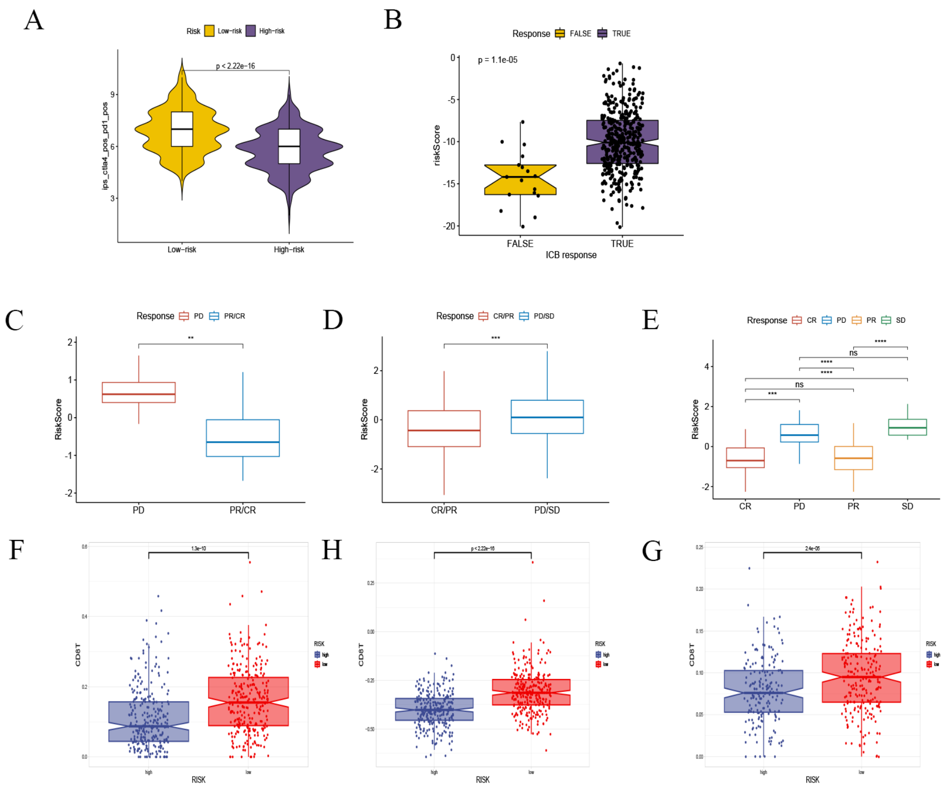

Supplementary Fig. 4

(A) Box plot of IPS between high-risk group and low-risk group

(B) Box plot of risk score between response group and non- response group

Box plot of risk score between PR/CR group and PD/SD group

(C) GSE78200

(D) imv210

(E) PRJEB23709

Box plot of CD8+ T cell between high-risk group and low-risk group

(F) CIBERSORT algorithm

(G) ssGSEA algorithm

(H) ImmuCellAI algorithm

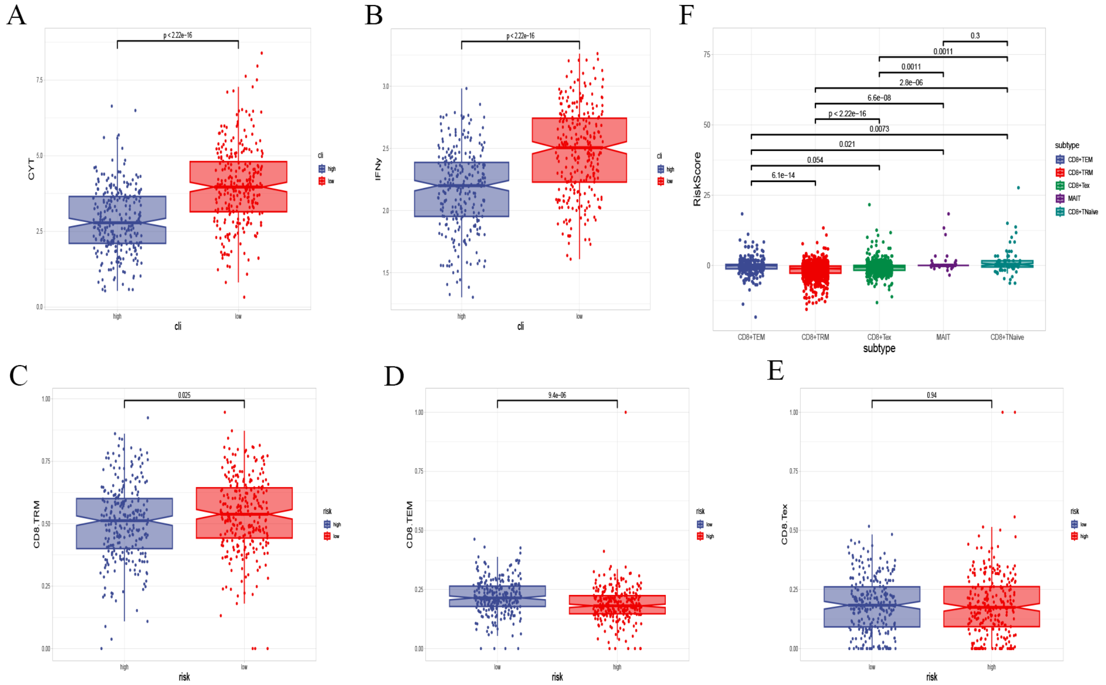

Supplementary Fig. 5

(A) Box plot of CYT score between high-risk group and low-risk group

(B) Box plot of IFN-γ score between high-risk group and low-risk group

Box plot showing the percent of CD8+ T cell subtype between high-risk group and low-risk group

(C) TRM

(D) TEM

(E) Tex

(F) Box plot showing the risk score between CD8+ T cell subtype

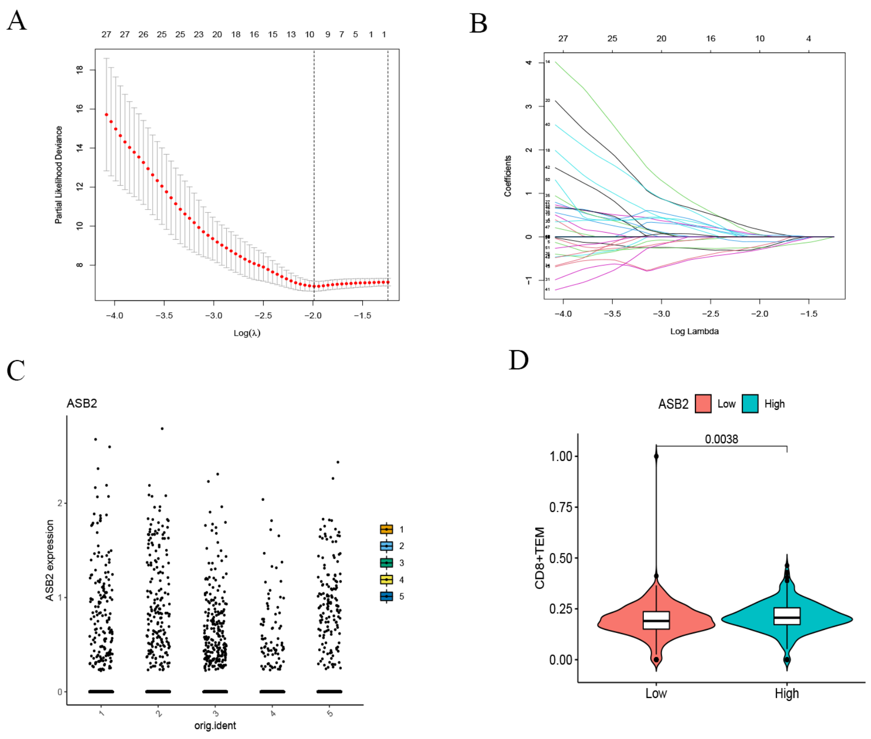

Supplementary Fig. 6

(A) Correlation plot between partial likelihood deviance and log regularization parameter (Log (λ))

(B) Situation of Coefficients when the partial likelihood deviance is minimized and the corresponding Log (λ) is determined

(C) Box plot showing the content of ASB2 in the single - cell dataset

(D) Box plot showing the content of ASB2 in TEM
